# Degeneration of Lumbar Intervertebral Discs: Characterization of Anulus Fibrosus Tissue and Cells of Different Degeneration Grades

**DOI:** 10.3390/ijms21062165

**Published:** 2020-03-21

**Authors:** Stefan Stich, Michal Jagielski, Anja Fleischmann, Carola Meier, Patricia Bussmann, Benjamin Kohl, Julia Schmidt, Jan-Philipp Krüger, Michaela Endres, Mario Cabraja, Kolja Reimann, Dominik Laue, Wolfgang Ertel, Michael Sittinger

**Affiliations:** 1 Department of Traumatology and Reconstructive Surgery, Campus Benjamin Franklin, Charité–Universitätsmedizin Berlin, Freie Universität Berlin, Humboldt-Universität zu Berlin and Berlin Institute of Health, 12203 Berlin, Germany; 2 Tissue Engineering Laboratory, BIH Center for Regenerative Therapies, and Department of Rheumatology and Clinical Immunology, Charité-Universitätsmedizin Berlin, Freie Universität Berlin, Humboldt-Universität zu Berlin and Berlin Institute of Health, 10117 Berlin, Germany; 3 Department of Spinal Surgery, Vivantes Auguste-Viktoria-Hospital, 12157 Berlin, Germany; 4 Research and Development Department, TransTissue Technologies GmbH, 10117 Berlin, Germany

**Keywords:** intervertebral disc, anulus fibrosus, degeneration, genome-wide microarray

## Abstract

Intervertebral disc (IVD) herniation and degeneration is a major source of back pain. In order to regenerate a herniated and degenerated disc, closure of the anulus fibrosus (AF) is of crucial importance. For molecular characterization of AF, genome-wide Affymetrix HG-U133plus2.0 microarrays of native AF and cultured cells were investigated. To evaluate if cells derived from degenerated AF are able to initiate gene expression of a regenerative pattern of extracellular matrix (ECM) molecules, cultivated cells were stimulated with bone morphogenetic protein 2 (BMP2), transforming growth factor β1 (TGFβ1) or tumor necrosis factor-α (TNFα) for 24 h. Comparative microarray analysis of native AF tissues showed 788 genes with a significantly different gene expression with 213 genes more highly expressed in mild and 575 genes in severe degenerated AF tissue. Mild degenerated native AF tissues showed a higher gene expression of common cartilage ECM genes, whereas severe degenerated AF tissues expressed genes known from degenerative processes, including matrix metalloproteinases (MMP) and bone associated genes. During monolayer cultivation, only 164 differentially expressed genes were found. The cells dedifferentiated and altered their gene expression profile. RTD-PCR analyses of BMP2- and TGFβ1-stimulated cells from mild and severe degenerated AF tissue after 24 h showed an increased expression of cartilage associated genes. TNFα stimulation increased MMP1, 3, and 13 expression. Cells derived from mild and severe degenerated tissues could be stimulated to a comparable extent. These results give hope that regeneration of mildly but also strongly degenerated disc tissue is possible.

## 1. Introduction

Low back pain is one of the most common diseases worldwide and its prevalence in the last few years has rapidly increased. It has a significant influence on patients’ life quality and an impact on the gross domestic product (GDP). Low back pain creates a severe economic burden in most countries [1,2]. Conservative therapies such as anti-inflammatory treatments or physiotherapy often only diminish the symptoms and relieve the patients’ pain [3], but cannot stop the progression of the disease. Thus, surgical interventions are often necessary. Degenerative changes in intervertebral discs (IVD) and disc herniations are common causes of lower back pain. In the case of herniation, fissures in the outer ring of the IVD, the anulus fibrosus (AF), evolve and lead to structural tears. The inner core of the IVD, the nucleus pulposus (NP), is pressed out and forms a prolapse, narrowing the spinal channel and compressing the nerves. This leads to pain and numbness. In the case of disc herniation, a standard surgical procedure is applied to remove the herniated NP tissue. However, the tear in the AF remains unrepaired. Therefore, a reherniation is a frequent problem with a reported rate of 23% [4]. Moreover, a loss of height of the intervertebral disc develops due to the loss of the herniated NP. This again enhances further degradation of the NP [5], leading to further neural foraminal stenosis. To avoid reherniation and to enable regenerative procedures of the NP, a closure of the ruptured AF area is necessary. Common methods are suture materials or mechanical devices, which have to be integrated and fixated in the intervertebral body [6,7]. However, using a solid barrier material to close AF ruptures can lead to loosening and dislocation of the material, resulting in irritation of spinal nerves and causing severe pain and the need for another surgical treatment [8]. The use of sutures for the closure of the AF might not be stable enough to resist the intradiscal pressure and the tensile forces that affect the IVD and the AF. Further, solid barriers or sutures can only maintain the status of the AF and do not improve healing and reversion of biomechanical changes that have occurred during herniation [9]. The AF mainly consists of proteoglycans and collagens, which have a low turnover rate [10,11]. Therefore, the AF has very limited self-healing potential, and the need of a regenerative therapeutic treatment is crucial. Regenerative approaches focus on the use of biomaterials with or without cells or the supplementation of growth or other factors to enhance extra cellular matrix production (ECM) of the cells [12,13]. Bone morphogenetic proteins (BMP) and other transforming growth factors (TGF) are prominent supplements in regenerative therapy research [14,15,16,17]. In addition, factors like chemokines or patient-derived blood and platelet compounds are potential candidates for regeneration [18,19].

Regardless of whether regenerative treatments use AF cells or factors that stimulate AF cells in vivo, it is important to analyze whether the cells still have a regenerative potential. Considering patients undergoing current therapies, the degeneration grade of the IVDs ranges from mild to severe. Therefore, the focus of our study lies in revealing the differences and similarities of these two groups. For annular cell-based therapies, earlier studies indicated that cells derived from AF tissues of different degeneration states are able to form transplants of sufficient quality in vitro after several weeks [18]. However, differences between tissues of different degeneration statuses and isolated cells are not sufficiently characterized, and their response on regenerative stimulators depending on the grade of degeneration remains unknown.

In this study, for the first time, transcriptomics data of human mild and severe degenerated AF tissues (classified according Pfirrmann score [20]) were compared and correlated to the corresponding transcriptomics data of isolated AF cells of passage 2. The Pfirrmann score is a magnetic resonance imaging score of the intervertebral disc and is divided into 5 grades. Grade 1 describes a healthy IVD with a bright white structure. Grade 2 shows a slight inhomogeneity with a normal disc height. In grade 3, signal intensity of the IVD decreases slightly and the structure may appear more gray. The disc height also may decrease slightly. In grade 4, the disc may appear gray to black and the distinction between AF and NP is lost. The disc height may be moderately to seriously decreased. In grade 5, IVD structure appears black and the disc space is collapsed [20]. In our study, we compared grade 2 or 3 discs as mild degeneration with grade 4 or 5 discs as severe degeneration. Further, glycosaminoglycan and collagen content of human AF tissues was compared. Finally, the response of cultivated human AF cells from different degenerated tissues on growth factors BMP2, TGFβ1, and the inflammatory cytokine TNFα were characterized using real-time detection polymerase chain reaction (RTD-PCR) 24 h after stimulation.

## 2. Results

### 2.1. Genome-Wide Microarray Gene Expression Profiling

**Figure 1:**
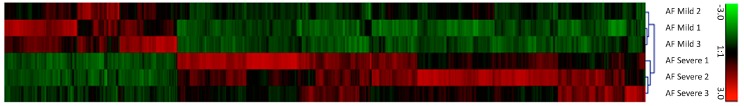
Genesis cluster analysis of native anulus fibrosus (AF) microarray signals from mild and severe degenerated AF.

Genome-wide microarray gene expression profiling revealed 788 differentially expressed genes with a present call in all samples in at least one of the two groups and a fold change (FC) of FC > 2 or FC < −2 when comparing native human AF tissues of mild versus severe degenerated IVDs. Of those expressed genes, 213 were more highly expressed in mild degenerated native tissue and 575 in severe degenerated AF tissue (Figure 1).

Included in the 213 genes more highly expressed in mild degenerated IVDs were cartilage associated genes such as *cartilage intermediate layer protein* (*CILP*, FC = 5.73), *decorin* (*DCN*, FC = 4.53), *aggrecan* (*ACAN,* FC = 4.32), *CILP 2* (FC = 4.26), *cartilage oligomeric matrix protein COMP,* FC = 3.45), *SRY* (*sex determining region Y*)*-box 9* (*SOX9*, FC = 2.71), *collagen, type II, alpha 1* (*COL2A1*, FC = 2.19), and *cartilage associated protein* (*CRTAP*, FC = 2.16). Included in the 575 genes more highly expressed in severe degenerated tissues were several bone associated genes such as *integrin-binding sialoprotein* (*IBSP*, FC = −34.78), *secreted phosphoprotein 1* (*SPP1*, FC = −26.17), *RUNX family transcription factor 2* (*RUNX2*, FC = −11.47), *periostin* (*osteoblast-specific factor*) (*POSTN*, FC = −9.78), *bone gamma-carboxcylglutamate protein* (*osteocalcin*) (*BGLAP* FC = −9.78), and different *matrix metalloproteinases* (*MMP*) such as *MMP2* (FC = −8.77), *MMP9* (FC = −21.28), *MMP13* (FC = −185.25), *MMP14* (FC = −5.36), and *MMP16* (FC= −9.85). After cell isolation and expansion up to passage 3, cells showed only 164 differentially expressed genes between the two groups, originating from mild and severe degenerated AF tissues with a present signal call in all samples in at least one of the two groups and a fold change of > 2 or < −2. From those 164 genes, 56 showed a higher expression in cells derived from mild degenerated AF, whereas 108 were more highly expressed in cells from severe degenerated tissue. Only 35 of these genes were also found among the 788 differentially expressed genes in native tissue (Table 1).

**Figure 2:**
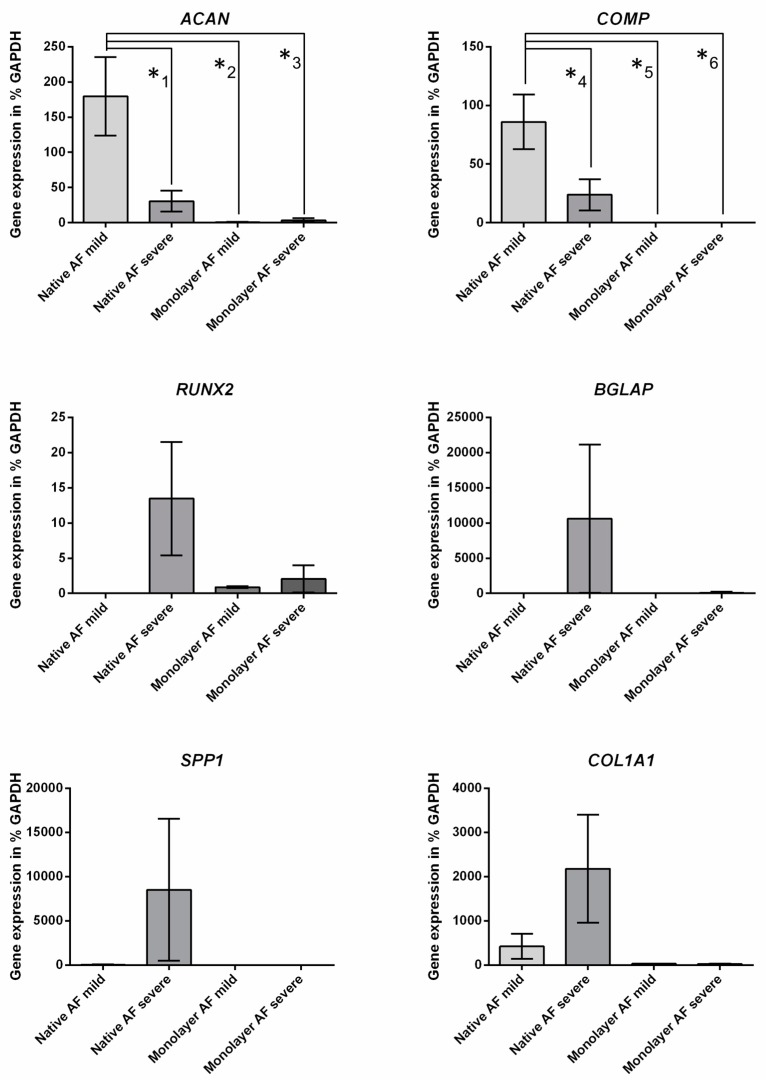
Real-time detection polymerase chain reaction (RTD-PCR) of chosen genes for validation microarray results of native AF tissue and monolayer cells from mild and severe degenerated intervertebral discs (IVDs). Significant differences in *aggrecan (ACAN)* and *cartilage oligomeric matrix protein (COMP*) gene expression (*^1^ *p* = 0.0260, *^2^ *p* = 0.0141, *^3^ *p* = 0.0141, *^4^ *p* = 0.0443, *^5^ *p* = 0.0114, *^6^ *p* = 0.0114) (error bars SEM).

Among the 35 genes that were differentially expressed in native tissue from degenerated IVDs and that were differentially expressed in monolayer expanded cells from AF tissue of differentially degenerated IVDs is *ACAN*. In native tissue, the expression was higher in mild degenerated AF. Monolayer culture revealed an opposite effect, the mean signal value dropped in both groups compared to native tissue but was higher in cells derived from severe degenerated AF. A list of all genes differentially regulated in native tissue and the monolayer is provided in the Supplementary Section (Tables S1 and S2). In order to verify microarray results, RTD-PCR was performed on the selected genes *ACAN*, *COMP*, *BGLAP*, *COL1A1*, *RUNX2*, and *SPP1*. *Glyceraldehyde-3-phosphate dehydrogenase* (*GAPDH*) was used as a housekeeping gene. *ACAN* showed a higher expression in native samples than in cell culture samples (Figure 2). In native tissue, *ACAN* was significantly more highly expressed in samples of mild degenerated tissues, whereas the expression in monolayer cultures was only on a very low level. The same results were found for the expression of *COMP*. No expression was seen in monolayer cultured cells. For *RUNX2*, *BGLAP*, *SPP1,* and *COL1A1* the highest expression was detected in native samples of severe degenerated IVD. In monolayer cells, only a minimal expression was detected in all samples. For native tissue with a mild degeneration, no expression of *RUNX2* and *BGLAP* was found.

### 2.2. Determination of Glycosaminoglycan and Collagen Content

**Figure 3:**
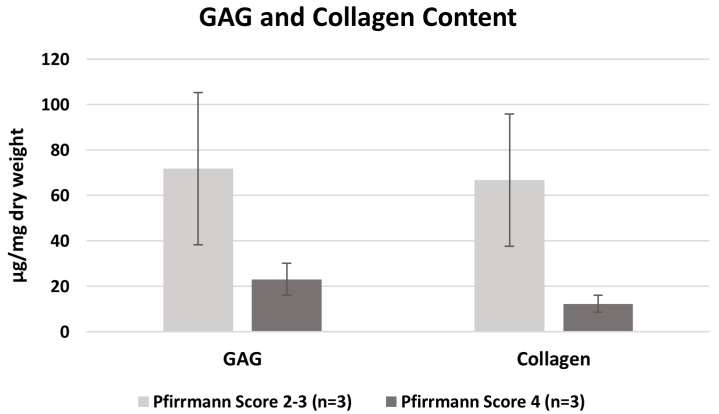
Glycosaminoglycan (GAG) and collagen content of mild (Pfirrmann score 2–3) and severe (Pfirrmann score 4) degenerated native annulus fibrosus (AF) tissues (error bars SEM).

The glycosaminoglycan (GAG) and the collagen content of native human AF tissue from mild and severe degenerated intervertebral discs were determined (*n* = 3 each). At 71.85 µg/mg dry weight (± 57.9), the GAG content in mild degenerated tissue was higher when compared to 23.13 µg/mg dry weight (± 12.2) in severe degenerated samples, but not significantly higher. Also, the collagen content of 66.76 µg/mg dry weight (± 50.5) was increased in mild degenerated samples compared to 12.33 µg/mg dry weight (± 6.4) in severe degenerated AF samples (Figure 3).

### 2.3. Stimulation Assay

**Figure 4:**
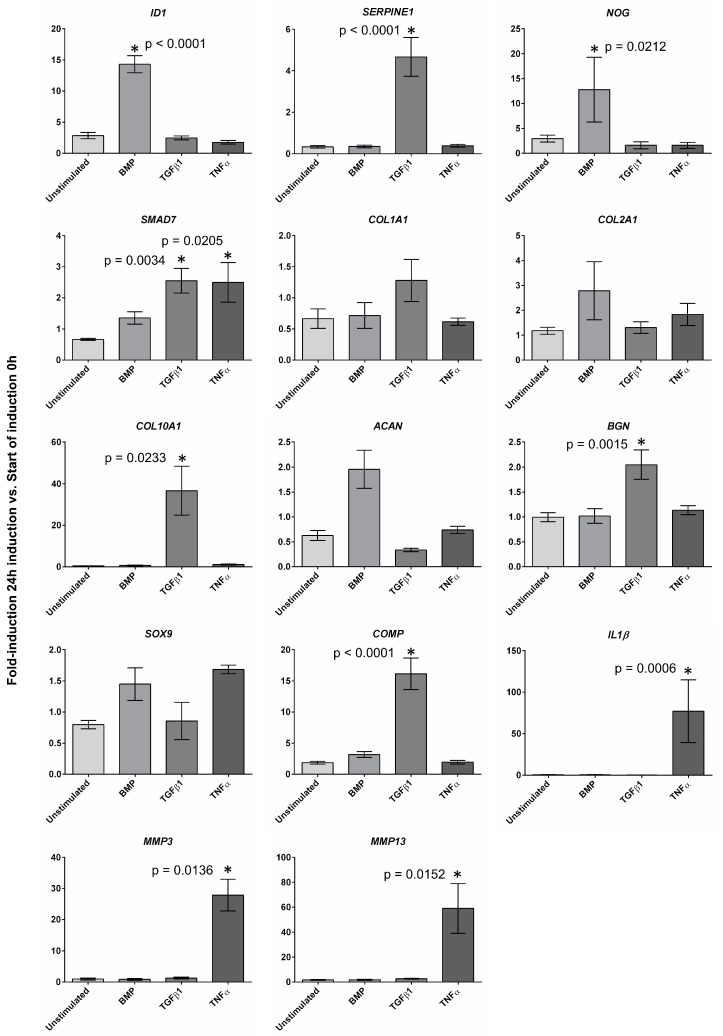
Gene expression of bone morphogenetic protein 2 (BMP2)-, transforming growth factor β1 (TGFβ1)-, and tumor necrosis factor α (TNFα)-stimulated cells and unstimulated cells after 24 h given as fold induction of gene expression at the starting point of the stimulation 0 h (error bars SEM); significant increase after 24 h indicated for * *p* < 0.05 and exact values given in the figure.

In order to test the immediate reaction upon stimulation of monolayer cultivated human AF cells after 24 h, the cells (*n* = 6; 3 from mild and 3 from degenerated AF tissues) were incubated with human recombinant BMP2 (5 nM), human recombinant TGFβ1 (200 pM), or human recombinant TNFα (10 ng/mL) (Figure 4). Stimulated and unstimulated cells after 24 h were compared to unstimulated day 0 controls and those at the fold induction of gene expression at the starting point of the induction 0 h (Figure 4). After stimulation of AF cells with BMP2, RTD-PCR revealed a significantly increased expression of *Inhibitor Of DNA Binding 1, HLH Protein* (*ID1*, *p* < 0.0001), and *noggin* (*NOG*, *p* = 0.0212), compared to unstimulated controls before stimulation (0 h). For *COL2* and *ACAN,* a higher mean expression was detected, but the results showed no statistical significance. Cell cultures stimulated with TGFβ1 showed after 24 h an increased expression of *serpin family E member 1* (*SERPINE1*, *p* < 0.0001), *biglycan* (*BGN*, *p* = 0.0015), *COMP* (*p* < 0.0001), and *COL10* (*p* = 0.0233) compared to unstimulated controls at the starting time (0 h). TGFβ1 stimulation of AF cells also resulted in increased mean values of *COL1A1* expression, but without statistical significance. Further, stimulation of AF cells with TNFα led to a significantly increased expression of *MMP3* (*p* = 0.0006), *MMP13* (*p* = 0.0136), and interleukin 1β (*IL1β*, *p* =0.0152) after 24 h compared to the unstimulated starting point (0 h). *SMAD Family Member 7* (*SMAD7*) was significantly stimulated by TGFβ1 (*p* = 0.0340) and TNFα (*p* = 0.0205). BMP2 and TNFα stimulation resulted in elevated mean values of *SOX9* expression but without statistical significance. Unstimulated cells after 24 h showed no increased expression of any tested gene compared to unstimulated cells at 0 h (Figure 4).

**Figure 5:**
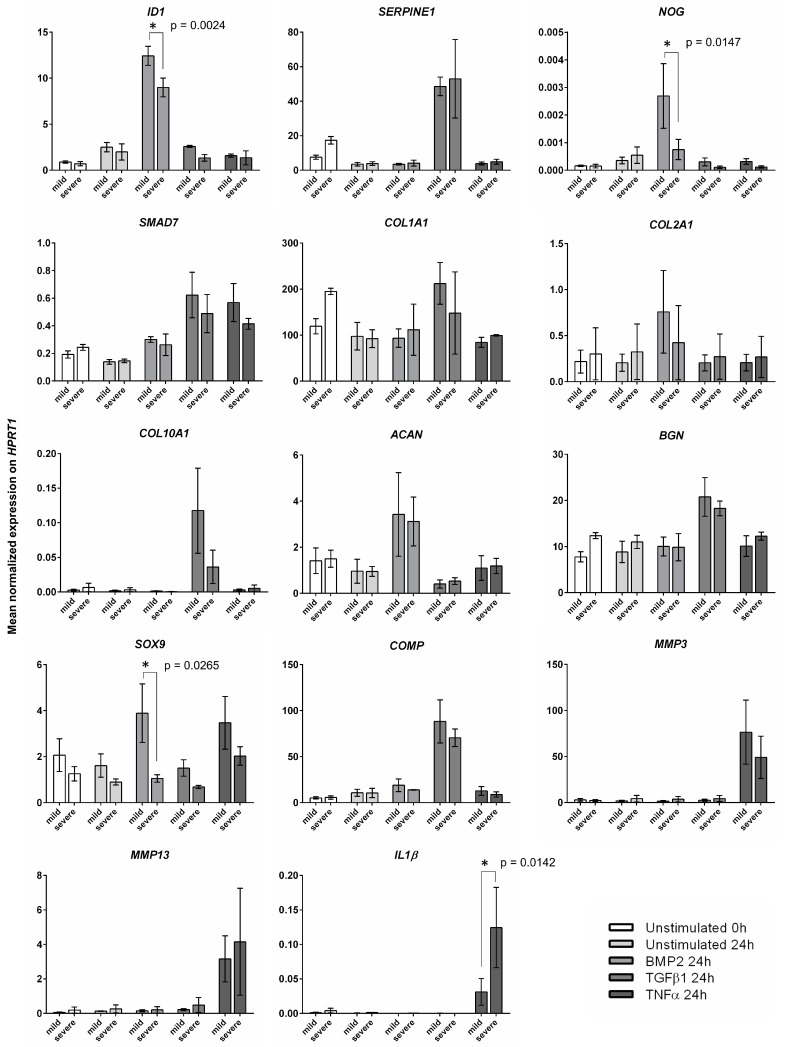
Mean normalized expression of bone morphogenetic protein 2 (BMP2)-, transforming growth factor β1 (TGFβ1)-, and tumor necrosis factor α (TNF α)-stimulated cells after 24 h and unstimulated cells at the starting point of 0 h and after 24 h (error bars SEM). * *p* < 0.05.

In a second step, we wanted to determine differences in gene expression after stimulation between cells derived from mild and severe degenerated AF tissues. Therefore, we separated our RTD-PCR results according to the degeneration state of their originating tissue. The mean normalized gene expression was referred to the house keeping gene *hypoxanthine phosphoribosyltransferase 1* (*HPRT1*) and compared between the cells from mild and severe degenerated AF tissues (Figure 5). In BMP2-stimulated cell cultures derived from mild tissues the gene expression was significantly higher for the genes *ID1* (*p* = 0.0046) and *NOG* (*p* = 0.0147) compared to samples derived from severe degenerated AF. The difference in *SOX9* gene expression between cells from mild and severe degenerated tissue was significant (*p* = 0.0265) (Figure 5), even if no significant overall change was seen compared to the unstimulated starting sample at 0 h (Figure 4). For TGFβ1 stimulated cells, there was no difference between cells from differently degenerated tissues. Only an elevated level in mean values for *COL10A1* was seen in cells from mild degenerated tissue, but without any statistical difference. TNFα stimulation lead to increased *IL1β* expression, significantly higher in the group derived from severe degenerated AF (*p* = 0.0142). MMP3 and MMP13 gene expression showed no significant changes in comparison to AF cells derived from differently degenerated tissues.

## 3. Discussion

The aim of our study was to investigate the capability of human AF tissue and AF derived cells to initiate regenerative therapy strategies for a possible future clinical application. The focus was placed on the analysis of differences of tissues from different grades of degeneration. Regenerative cell–based therapies such as tissue engineering and application of growth factors or other stimulants depend on the ability of cells to produce repair tissue either ex vivo for transplantation or in vivo for self-repair. It is utterly unknown whether relevant cells from severely degenerated tissues are actually capable of being stimulated towards a regenerative metabolism. Native AF tissue is a heterogeneous tissue. Especially during degeneration, an ingrowth of blood vessels and nerve roots can occur. It was our aim to compare the whole gene expression of native AF as a whole heterogeneous tissue and to see the changes between mildly and severely degenerated AF. For cell isolation and expansion we used established protocols based on enzymatic digestion of the AF and cell expansion of plastic-adherent cells. Non-adherent cell types were washed out and the cells from all tissues showed a homogeneous fibroblast-like morphology as demonstrated before [19]. Since our overall goal was a regenerative approach, we focused on cells with a chondrogenic potential. In previous experiments we were able to demonstrate that AF cells isolated with the used enzymatic procedures were able to show this chondrogenic potential [17,18,19].

We used genome-wide Affymetrix Human Genome U133A Plus 2.0 array to assess the differences between human lumbar AF tissues from mild and severe degenerated IVDs according to the Pfirrmann grading [20]. Our results showed 788 genes differentially expressed between mild (Pfirrmann score 2–3) and severe degenerated AF tissue (Pfirrmann score 4–5) (detection 100% in cells with higher expression; fold change ≥ 2 or ≤ −2) with 213 genes more highly expressed in mild and 575 genes in severe degenerated AF tissue. Among the genes more highly expressed in mild degenerated tissues, typical cartilage associated genes *ACAN*, *DCN*, *COMP*, *COL2A1*, and *SOX9* were found. The most common proteoglycan in IVD tissue is aggrecan. In degeneration processes, aggrecan is degraded by the MMPs and aggrecanases [21]. It is known from other studies that degenerated IVD tissue contains several MMPs including MMP1, MMP2, MMP3, MMP7, MMP8, MMP-9, and MMP13 [21,22]. Our findings in genome-wide microarray expression analysis found *MMP2, MMP9*, and *MMP13* from this list and additionally *MMP14* and *MMP16* with increased gene expression in severe degenerated tissues. In addition, we found *DCN* more highly expressed in mild degenerated AF tissue. Decorin plays a major part in the composition of the IVD matrix and is the most prominent member of the small proteoglycan family in AF [23,24]. In our study, proteoglycan content of AF tissues from mild and severe degenerated IVD was compared, and an increased, but not significant, mean level of GAG was found. The loss of proteoglycan is significant in degeneration processes in the IVD. Large aggrecan molecules are fragmented and smaller fragments diffuse out of the IVD. Due to the GAG loss, the IVD matrix loses its capability to contain water, leading to a loss of hydration [22]. Furthermore, the amount of collagen was higher but not significant in mild degenerated AF tissues. In microarray gene expression analysis, we found *COL2A1* more highly expressed in mild degenerated AF tissues and *COL1A1* more highly expressed in severe degenerated tissues. In native AF tissue, the collagen type I content in the inner AF region was lower compared to the outer AF region. For collagen type II, it was the opposite [25]. Further, the cartilage specific genes for *COMP* and the transcription factor *SOX9* were found in mild degenerated tissue. *COMP* is described to be highly expressed in AF and the protein functions of COMP include binding other matrix proteins and catalyzing polymerization of type II collagen fibrils [26]. This hints that during degenerative AF processes, these functions might be decreased. The decrease of *SOX9* supports the hypothesis of a reduced cartilage-like AF tissue synthesis [27]. In AF derived from severe degenerated IVDs, genes associated with osteogenesis and ossification were found (*IBSP*, *SPP1*, *RUNX2*, *POSTN*, and *BGLAP*). Since all potential bone fragments were removed during preparation of IVD tissues, these findings indicate a potential beginning of ossification of the AF tissue during degeneration. These results correlate with clinical observations. Patients with lumbar intervertebral osteochondrosis develop disc space narrowing and osteophyte formation, so called spondylophytes [28]. Comparing the generated results from native tissue with microarray data from monolayer cultured cells derived from the same tissues, a down regulation of highly expressed genes was seen that indicates a general de-differentiation of the cells. The difference in gene expression between cells derived from mild and severe degenerated AF diminished and a conversion occurred. Only 164 genes were differentially expressed. Among them, only 35 genes were already differentially expressed in native tissue. Most of these genes showed a lower signal intensity than in native tissue or reduced the FC, e.g., the chemokine *CXCL12* (native tissue FC = −5.53, signal intensity mild = 461.27, signal intensity severe = 2751.70; monolayer FC = −4, signal intensity mild = 317.13, signal intensity severe = 1576.40). Other genes demonstrated a reversed gene expression. The gene *ACAN,* coding for the proteoglycan aggrecan, was one of them. Here, the signal intensity was highly reduced from 6901.30 (mild) and 2063.57 (severe) to 161.73 (mild) and 410.97 (severe) and the FC changed from 4.32 to −2.35. This indicates the possibility of dedifferentiation processes in monolayer cultures [29]. Another possibility of the reversed FC in monolayer culture compared to native AF tissue is due to the heterogeneity of the native tissue. Especially during degeneration, an ingrowth of blood vessels and nerve roots can occur. Due to cell isolation and culture conditions, the growth of cells with a chondrogenic differentiation potential is promoted [17,18,19]. That might explain the changes in monolayer gene expression. Nevertheless, another possibility might be the sensitivity of the microarray. In most cases of the reversed FC, one of the FCs was close to 2 or respectively −2, indicating significant FCs. For *ACAN,* with an FC of 4.32 in native tissues and an FC of −2.35 in monolayer cells, the corresponding signals decreased in mild and severe degenerated samples in monolayer cells compared to native tissue as mentioned above (from 6901.30 (mild) and 2063.57 (severe) in native AF to 161.73 (mild) and 410.97 (severe) in monolayer). A RTD-PCR validation confirmed this decrease, but a significant difference between monolayer cells from mild and severe AF was not seen. According to the manufacturer’s protocols, microarray sensitivity is 1:100,000. The technique used for microarrays is based on hybridization. The sensitivity for a real-time PCR is 1000-fold higher [30]. Further, the microarray is based on one reference sequence (e.g., *ACAN*: NM_001135). For real-time PCR, the TaqMan primer covers more alternative reference sequences (e.g., *ACAN*: NM_001135, NM_013227, XM_011521313, and XM_011521314).

In order to further validate the microarray results, RTD-PCR was used for other selected disc-cartilage-associated genes. For *COMP,* the microarray results were also confirmed. A significantly higher gene expression was found in native tissues from mild degenerated AF compared to native tissue from severe degenerated AF and monolayer expanded cells. For the genes *RUNX2*, *BGLAP*, *SPP1*, and *COL1A1,* higher but not significant mean values were found in native tissue compared to those of severe degenerated AF. For *RUNX2* and *BGLAP*, a gene expression in native tissue from mild degenerated AF could not be detected. These results confirm the microarray results and support the theory of a potential dedifferentiation in monolayer cultures [29].

In order to see if monolayer expanded cells are still responsive for induction with the growth factors BMP2 and TGFβ1 and therefore applicable for a potential clinically relevant regenerative therapy, a 24 h induction assay was performed (Figure 4). The genes involved in BMP (*ID1*, *NOG*) and TGF (*SERPINE1*, *SMAD7*) pathways as well as genes for collagens (*COL1A1*, *COL2A1*, and *COL10A1*) and proteoglycans (*ACAN*, *BGN*) and other extracellular matrix proteins and transcription factors (*SOX9*) were selected to assess the influence of the growth factors. Furthermore, a TNFα induction was applied to induce degenerative procedures. *ID1* and *NOG* were significantly induced by BMP2 and *SERPINE1,* and *SMAD7* expression was significantly increased upon TGFβ1 stimulation. *SMAD7* was also significantly regulated by TNFα stimulation. Smad7 is a TGFβ1 antagonist of TGFβ1 signaling [31] and is involved in TNFα-mediated processes. TNFα/NF-κB induced Smad7 suppresses TGFβ/SMAD signaling [32].

Among the selected collagens, after 24 h, only *COL10A1* expression was significantly regulated by TGFβ1 stimulation. Since collagens such as collagen type I and II are generally only produced during later chondrogenic stages, we might only see a slight increase but not significant induction of *COL1A1* by TGFβ1 and *COL2A1* by BMP2 after 24 h [33,34]. TGFβ1 significantly induced genes coding for extracellular matrix components such as BGN and COMP in gene expression. *ACAN* only showed a higher mean value 24 h after BMP2 stimulation but no significant induction. The gene expression of the transcription factor *SOX9* was not significantly changed after 24 h by any used stimulant. Furthermore, a TNFα induction was described to induce degenerative procedures. *MMP3*, *MMP13*, and *IL1β*, which all play an important role in IVD degeneration, were selected to confirm TNFα stimulation [35]. The gene expression of all three genes was significantly induced by TNFα.

After the successful stimulation of these AF cell cultures, we took a closer look at how cells from differently degenerated AF tissues reacted to the stimuli BMP2, TGFβ1, and TNFα (Figure 5). Stimulation with TGFβ1 showed no significant differences in gene expression regarding the degeneration grade of the AF cell source. BMP2 stimulation revealed a significantly higher gene expression of *ID1*, *NOG*, and *SOX9* after 24 h in cells derived from mild degenerated AF. Further, TNFα stimulation led to a significantly higher gene expression of *IL1β* in cell cultures derived from severe degenerated AF tissue. These differentially expressed genes after 24 h of induction may indicate a lower capacity towards cell differentiation and a tendency to a higher inflammatory response in cells derived from severe AF tissue. Nevertheless, differentiation studies producing 3D cell cultures with TGF and BMP stimulation over several weeks demonstrated AF grafts with similar properties independent of AF degeneration grade [18]. This may indicate that our findings after 24 h demonstrate a delayed reaction of AF cells from severe degenerated tissues upon this stimulation with TGF and BMP. Another possibility is that AF cells from severe degenerated tissues could need slightly higher doses of TGF and BMP to obtain the same reaction as AF cells from mild degenerated tissues. Nevertheless, despite minor differences, we could demonstrate that even cells from severe degenerated AF tissues were able to show a response after stimulation.

Taken together, our results demonstrated that gene expression in native AF tissue was different considering the degeneration grade of the IVD. During monolayer cultivation the gene expression was more equalized. However, a clear limitation of this study is the low number of specimens used in the experiments. In order to obtain highly significant RTD-PCR results, a larger number of specimen is beneficial. More specimens can help to compensate for potential outliers, leading to more reliable results. Also, for microarray experiments, an increase of AF samples would potentially lead to a more distinct gene list. Candidate genes at the edge of significance with a low FC of slightly over 2 or under −2 could be excluded. However, the genes included in our evaluation in native AF tissue *COMP* (FC = 3.45), *ACAN* (FC = 4.32), and *SOX9* (FC = 2.71) have a stable higher FC and *RUNX2* (FC = −11.47), *BGLAP* (FC = −9.78), and *MMP13* (FC = −185.25) have a stable lower FC.

Finally, cells from tissues of all degeneration grades showed a response to stimulation compared to non-stimulated controls. Therefore, even cells from severe degenerated AF demonstrated that they are suitable for future clinical application in regenerative therapies. Prior to a clinical application of cells or factors to stimulate AF cells in patients, in vivo studies have to be performed. A potential application is the injection of a cell/factor loaded hydrogel into an IVD defect. The disadvantage is a potential leakage or a new disruption due to a constant mechanical loading. Also, scaffold-based regeneration approaches are possible, but the problem of pressure and mechanical loading are still present. A combination of an injectable formulation for regeneration in a ruptured AF with a mechanical sealing of the defect by a slow degrading device seems to be promising. The sealing scaffold must stay in place until cells and/or factors have initiated a successful stable regeneration of the tissue underneath. Until a prospective in vivo application is feasible, more in vivo evaluations have to be performed. Among them, cell numbers for in vivo treatments have to be determined and an in vivo safety and proof of principle have to be demonstrated in preclinical studies. In the case of a factor-based regenerative approach, effective doses and lethal doses have to be evaluated in vivo. So far, our study is just the beginning of the development chain, giving proof that cells from mildly and severely degenerated AF are able to initiate a regenerative process in vitro.

## 4. Materials and Methods

**Figure 6:**
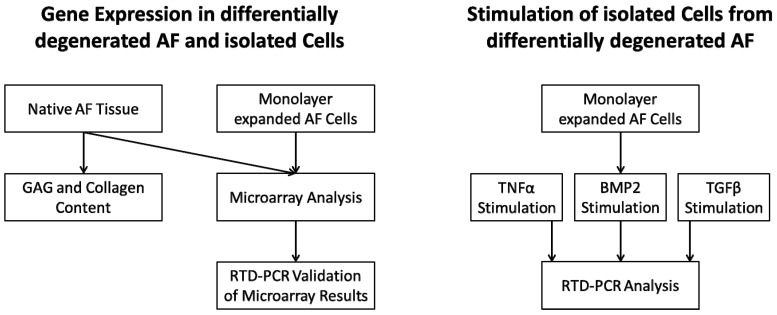
Experimental design for gene expression in differentially degenerated anulus fibrosus (AF) and isolated cells, and for the stimulation of isolated cells from differentially degenerated AF.

All experiments were performed according to the experimental design (Figure 6).

### 4.1. Sample Preparation for Genome-Wide Microarrays

Native human AF tissues of three mild and three severe degenerated IVDs were snap frozen in liquid nitrogen and stored at −80 °C until ribonucleic acid (RNA) isolation. Cell isolation from native AF tissues of three mild and three severe degenerated IVDs was performed by overnight digestion using a collagenase mix as described earlier [19]. Cells were seeded with a density of 10,000 cells/cm^2^ and cultivated up to passage 2. Cell lysis of the tissue was performed using Tri-Reagent (Sigma-Aldrich, Taufkirchen, Germany). For native tissue, the tissue was minced in Tri-Reagent using the Qiagen TissueRuptor (Qiagen, Hilden, Germany). After 1-bromo-3-chloropropane extraction (Sigma-Aldrich), further RNA isolation was performed using the Qiagen RNeasy Mini Kit (Qiagen, Hilden, Germany) according the manufacturer’s protocol.

### 4.2. Genome-Wide Microarray Gene Expression Profiling

Total RNA of native human tissue and expanded cells in passage 3 of three mild and of three severe degenerated AF tissues was used for genome-wide microarray analysis with the Affymetrix HG-U133 plus 2.0 array (Affymetrix, Santa Clara, CA, USA) according to the manufacturer’s recommendations. In brief, 2 μg of total RNA was used to synthesize biotin-labeled cRNA and 10 μg of fragmented cRNA were then hybridized to gene chips at 45 °C for 16 h. After washing and staining, gene chips were scanned with the GeneArray scanner controlled by Affymetrix GCOS 1.4 software (Affymetrix, Santa Clara, CA, USA). Raw gene expression data were processed and normalized with the Affymetrix GCOS 1.4 software and by robust multichip average (RMA) [36]. All samples from the mild degenerated group were compared to all samples from the severe degenerated group. The same tests were performed for the respective cell culture groups. Genes that showed differential expression (> 2 fold induction or repression) in at least 7 out of 9 comparisons and showed a positive detection call indicated by the GCOS 1.4 software in one of the groups were selected for further analysis. Expression of selected genes was verified by RTD-PCR. Hierarchical clustering with normalized gene expression values was performed with Genesis software v.1.8.1 (A.Sturn & R.Snajder, Graz University of Technology, Graz, Austria) [37].

### 4.3. Determination of Glycosaminoglycan (GAG) and Collagen Content

In order to determine sGAG and collagen content of native human AF samples, the tissues were snap-frozen in liquid nitrogen and stored at −80 °C until used. To measure dry content, native tissues were dried using phosphorus pentoxide (Roth, Karlsruhe, Germany) in a desiccator. After 9 h, dried tissues were weighed. For sGAG and collagen content determination, AF tissues were digested with proteinase K (Sigma-Aldrich, Taufkirchen, Germany) solution (10 mg/mL dissolved in 50 mM Tris/HCl, 1 mM ethylenediaminetetraacetic acid (EDTA), 0.5% Tween 20, pH 8.5) for 16 h at 65 °C and 500 rpm shaking. After 30 min centrifugation at 10,000× *g*, the supernatant was stored at 4 °C. For GAG content determination, a standard dimethyl methylene blue (DMMB) assay was used [38]. Samples were diluted in phosphate-buffered EDTA (100 mM Na_2_HPO_4_ and 5 mM EDTA, pH8) and DMMB solution (8.9 mM DMMB hydrochloride in 600 mg glycine, 467 mg NaCl, and 200 mL distilled water) was added. The absorption at 525 nm was immediately measured. As a standard, chondroitin sulfate (Sigma-Aldrich, Taufkirchen, Germany) was used for calculation of GAG content.

The quantification of the o-hydroxyproline content was performed using a colorimetric reaction of the samples with chloramine T and diaminobenzaldehyde against an o-hydroxyproline standard curve [39]. After incubation with proteinase K, a hydrolysis of the supernatant with HCl (6 mol/L) (1:2) was performed at 105 °C for 24 h using a hybridization oven. Hydrolysates (50 µL) were neutralized with 450 μL NaOH-citrate/acetate buffer (pH 5.4; Roth, Karlsruhe, Germany). To the 500 µl, an amount of 250 µL Chloramine-T reagent (Merck) was added to each well. After shaking, samples settled for 20 min at 22 °C. An amount of 250 μL of perchloric acid (6.2 mol/L; Roth, Karlsruhe, Germany) was added and samples were allowed to settle for 12 min at 22 °C. Then, 250 μL of p-dimethyl-amino-benzaldehyde (Avantor, Radnor, PA, USA) were added, the solution was shaken at 400 rpm at 60 °C for 20 min. The absorption was measured at 565 nm using a Genios spectral photometer (Tecan, Männedorf, Switzerland). Each measure included hydroxyproline (AppliChem, Darmstadt, Germany) dilution series standards and blank values of the citrate/acetate buffer. In order to calculate the collagen content, the conversion factor of 1 g of hydroxyproline per 7.1 g of collagen was used [40].

### 4.4. Stimulation Assay

Human AF cells were isolated enzymatically and cultivated in cultivation medium containing 10% fetal bovine serum (FBS) up to passage 3. Then, cells were seeded in 6-well plates, 350,000 cells in each well. Cells were allowed to adhere for 24 h. Then, the medium was changed and cells were starved for 3 h in a minimal medium (same as cultivation medium, but with 0.5% FBS). Afterwards, one of the following stimulants was added to the medium: human recombinant BMP2 (5 nM) [41] (R&D Systems, Minneapolis, MN, USA), human recombinant TGFβ1 (200 pM) [42] (Peprotech, Hamburg, Germany), or TNFα (10 ng/mL) [43] (Peprotech, Hamburg, Germany). Before induction and after 24 h, cells were lysed using Qiagen lysis buffer (Qiagen, Hilden, Germany) supplemented with β-mercaptoethanol (Roth, Karlsruhe, Germany). Lysed samples were stored at −80 °C until isolation. Isolation of mRNA was accomplished using the Qiagen RNeasy Mini Kit (Qiagen, Hilden, Germany) according to manufacturer’s protocol.

### 4.5. RTD-PCR

For complementary deoxyribonucleic acid (cDNA) synthesis of isolated RNA, the QuantiTect Reverse Transcription Kit (Qiagen, Hilden, Germany) was used. For each sample, 1 µg of total RNA was transcribed. First, remnants of genomic DNA (gDNA) were removed by the gDNA wipeout buffer. The cDNA synthesis was performed according to manufacturer’s protocol, resulting in a cDNA concentration of 50 ng/µL.

TaqMan-based (Thermo Fisher Scientific, Waltham, MA, USA) RTD-PCRs were completed for selected genes *ACAN*, *COMP*, *BGLAP*, *collagen type I* (*COL1*), *RUNX2,* and *SPP1* of the genome-wide microarray gene expression. The RTD-PCR was performed according to manufacturer’s protocols using a Step One Plus real-time PCR cycler (Thermo Fisher Scientific, Waltham, MA, USA). TaqMan Assay IDs are given in Table 1.

RTD-PCR using SYBR Green (Thermo Fisher Scientific, Waltham, MA, USA) systems were performed for selected genes in the cell-stimulation assay. In brief, amplification 10 ng cDNA was done using a Step One Plus real-time PCR cycler (Thermo Fisher Scientific, Waltham, MA, USA) under the conditions of 10 min at 95 °C, then for 36 cycles of 15 s at 95 °C, 1 min at 60 °C, followed by 4 °C cooling. Melting curve was determined under the following conditions: 95 °C for 15 s, 60 °C for 1 min, followed by an increase of temperature in 0.3 °C steps up to 95 °C with 15s remaining on each temperature step. Primer sequences are given in Table 2.

Statistical significance analyses for the comparison of the gene expression of induced vs. noninduced samples were performed by using one-way ANOVA with Holm–Sidak’s multiple comparison test. For analyses of differences of mild and severe groups, two-way ANOVA with Holm–Sidak’s multiple comparison test was used.

## 5. Conclusions

Our findings suggest that gene expression of AF cells in human native tissues varies depending on degeneration grade. Common cartilage-associated genes were significantly more highly expressed in AF tissues from mild degeneration grades, and genes known from ossification processes were more highly expressed in severe degenerated AF tissues. During in vitro cultivation and expansion, the cells dedifferentiated and gene expression profiles of cells from mild and severe degenerated AF tissues converged. All cell cultures responded to stimulation with BMP2, TGFβ1, and TNFα. Cells from mild degenerated tissue responded to BMP2 with significantly higher gene expression of *ID1*, *NOG*, and *SOX9*. TNFα stimulation led to a higher response in *IL1β* gene expression in cells derived from severe degenerated tissues. Nevertheless, cells from tissues from all degeneration grades showed a response to stimulation compared to non-stimulated controls and demonstrated that they might be suitable for future clinical application in regenerative therapies.

## Figures and Tables

**Table 1:** Genes with different expressions in both mild vs. severe degenerated anulus fibrosus (AF) tissue (NA) and in monolayer cells (ML) from mild vs. severe degenerated AF tissue (35 genes).

Affymetrix ID	Symbol	Accession No.	Mean Signal NA Mild	Mean Signal NA Severe	Native FC	Mean Signal ML Mild	Mean Signal ML Severe	Monolayer FC	Name
205679_x_at	*ACAN*	NM_001135	6901	2063	4.32	161	410	−2.35	*aggrecan*
222608_s_at	*ANLN*	NM_018685	34	153	−4.19	851	320	2.76	*anillin, actin binding protein*
227911_at	*ARHGAP28*	NM_001010000	56	168	−3.51	160	354	−2.32	*Rho GTPase activating protein 28*
219087_at	*ASPN*	NM_017680	20,240	8186	2.35	56	558	−9.40	*asporin*
218899_s_at	*BAALC*	NM_001024372	653	154	5.12	238	87	2.66	*brain and acute leukemia, cytoplasmic*
207172_s_at	*CDH11*	NM_001797	118	810	−7.24	931	2050	−2.05	*cadherin 11, type 2, OB-cadherin (osteoblast)*
204170_s_at	*CKS2*	NM_001827	132	362	−2.13	1146	508	2.14	*CDC28 protein kinase regulatory subunit 2*
211343_s_at	*COL13A1*	NM_001130103	205	1454	−5.61	288	89	2.94	*collagen, type XIII, alpha 1*
225647_s_at	*CTSC*	NM_001114173	101	471	−4.78	1430	547	2.35	*cathepsin C*
203666_at	*CXCL12*	NM_000609	461	2751	−5.53	317	1576	−4.00	*chemokine (C-X-C motif) ligand 12 (stromal cell-derived factor 1)*
201289_at	*CYR61*	NM_001554	10,725	5011	2.37	3354	7457	−2.19	*cysteine-rich, angiogenic inducer, 61*
202481_at	*DHRS3*	NM_004753	697	266	2.46	395	1167	−2.52	*dehydrogenase/reductase (SDR family) member 3*
226281_at	*DNER*	NM_139072	1458	497	5.00	828	355	3.10	*delta/notch-like EGF repeat containing*
232204_at	*EBF1*	NM_024007	300	686	−2.06	276	573	−2.13	*early B-cell factor 1*
219134_at	*ELTD1*	NM_022159	348	960	−3.38	110	46	2.41	*EGF, latrophilin and seven transmembrane domain containing 1*
40665_at	*FMO3*	NM_001002294	38	173	−3.68	61	135	−3.59	*flavin containing monooxygenase 3*
202709_at	*FMOD*	NM_002023	24,906	7901	3.15	273	592	−2.54	*fibromodulin*
204984_at	*GPC4*	NM_001448	79	433	−4.67	126	277	−2.08	*glypican 4*
209170_s_at	*GPM6B*	NM_001001994	201	573	−5.92	121	336	−3.25	*glycoprotein M6B*
214091_s_at	*GPX3*	NM_002084	11,915	1856	5.79	1232	2315	−3.27	*glutathione peroxidase 3 (plasma)*
227314_at	*ITGA2*	NM_002203	133	450	−2.46	267	114	2.18	*integrin, alpha 2 (CD49B, alpha 2 subunit of VLA-2 receptor)*
219949_at	*LRRC2*	NM_024512	279	116	2.64	29	66	−2.42	*leucine rich repeat containing 2*
226210_s_at	*MEG3*	NR_002766	560	161	4.13	121	51	2.19	*maternally expressed 3 (non-protein coding)*
203434_s_at	*MME*	NM_000902	32	109	−3.17	126	444	−3.25	*membrane metallo-endopeptidase*
227394_at	*NCAM1*	NM_000615	68	1034	−5.12	84	27	3.17	*neural cell adhesion molecule 1*
218039_at	*NUSAP1*	NM_001129897	82	214	−2.46	572	178	2.83	*nucleolar and spindle associated protein 1*
213125_at	*OLFML2B*	NM_015441	386	2864	−6.96	530	1360	−2.35	*olfactomedin-like 2B*
204040_at	*RNF144A*	NM_014746	211	525	−2.64	120	246	−2.00	*ring finger protein 144A*
209773_s_at	*RRM2*	NM_001034	31	159	−4.32	891	203	3.48	*ribonucleotide reductase M2*
204051_s_at	*SFRP4*	NM_003014	496	2665	−5.92	1749	5272	−3.43	*secreted frizzled-related protein 4*
229151_at	*SLC14A1*	NM_001128588	1420	253	4.59	854	128	10.00	*solute carrier family 14 (urea transporter), member 1 (Kidd blood group)*
225987_at	*STEAP4*	NM_024636	447	1962	−3.22	1030	1855	−2.19	*STEAP family member 4*
213247_at	*SVEP1*	NM_153366	107	511	−4.09	316	1184	−3.46	*sushi, von Willebrand factor type A, EGF and pentraxin domain containing 1*
209228_x_at	*TUSC3*	NM_006765	136	299	−2.54	1121	614	2.00	*tumor suppressor candidate 3*
230083_at	*USP53*	NM_019050	1201	333	4.13	213	411	−2.18	*ubiquitin specific peptidase 53*

**Table 2:** Gene accession numbers, TaqMan assay numbers or primer sequences, and expected transcript lengths used for the detection of gene expression.

Gene	EMBL Accession No.	Sequence (5′-3′)/ Thermo Fisher Scientific Assay No.	PCR Product Size (bp)
*GAPDH*	NM_002046	Cat. No. Hs99999905_m1	122
*ACAN*	NM_001135	Cat. No. Hs00153936_m1	91
*COMP*	NM_000095	Cat. No. Hs00164359_m1	101
*RUNX2*	NM_001015051	Cat. No. Hs00298328_s1	124
*BGLAP*	NM_199173	Cat. No. Hs00609452_g1	74
*SPP1*	NM_000582	Cat. No. Hs00167093_m1	65
*COL1A1*	NM_000088	Cat. No. Hs01076780_g1	109
*HPRT1*	NM_000194	F: CTTTGCTGACCTGCTGGATT R: CTGCATTGTTTTGCCAGTGT	211
*ID1*	NM_002165	F: GCTGCTCTACGACATGAACG R: CCAACTGAAGGTCCCTGATG	131
*SERPINE1*	NM_000602	F: CTTTCAGACCAAGAGCCTCTC R: CCATGCGGGCTGAGACTAT	118
*SOX9*	NM_000346	F: GCAAGCTCTGGAGACTTCTGA R: CTGCAGCGCCTTGAAGAT	206
*NOG*	NM_005450	F: GTGCAAGCCGTCCAAGTC R: GCTAGAGGGTGGTGGAACTG	219
*SMAD7*	NM_00119082	F: GGCTTTCAGATTCCCAACTTCTT R: ATTTTGCTCCGCACCTTCT	217
*COL1A1*	NM_000088	F: GGCAACGATGGTGCTAAGG R: GACCAGCATCACCTCTGTCA	139
*COL2A1*	NM_001844	F: GATGGCTGCACGAAACATACC R: AAGAAGCAGACCGGCCCTAT	155
*COL10A1*	NM_000493	F: CACCATAAAGAGTAAAGGTATAGCAGT R: GCACACCTGGTTTCCCTACA	194
*COMP*	NM_000095	F: CCAACTCAAGGCTGTGAAGTC R: GTCCTTCCAACCCACGTTTC	130
*ACAN*	NM_001135	F: GACACCCCATGCAATTTGAGAA R: CCGCACCAGGGAATTGATCT	242
*BGN*	NM_001711	F: CTCCCAGACCTCAAGCTCC R: GGGGTTGTTGAAGAGGCTGAT	138
*IL1β*	NM_000576	F: CCCTAAACAGATGAAGTGCTCC R: AGAAGGTGCTCAGGTCATTCTC	197
*MMP3*	NM_002422	F: CTATCAGAGGAAATGAGGTACGAGC R: GCCTGGCTCCATGGAATTTC	179
*MMP13*	NM_002427	F: CCCCAGGCATCACCATTCAA R: CAGGTAGCGCTCTGCAAACT	150

## Supplementary Materials

Supplementary materials can be found at https://www.mdpi.com/1422-0067/21/6/2165/s1. Table S1: Differentially regulated genes in native AF tissue of IVDs with mild and severe degeneration grades; Table S2: Differentially regulated genes in monolayer cultivated cells derived from AF tissue of IVDs with mild and severe degeneration grades.

## Abbreviations

ACAN	Aggrecan
AF	Anulus fibrosus
BGLAP	Bone gamma-carboxcylglutamate protein (osteocalcin)
BGN	Biglycan
BMP	Bone morphogenetic protein
CILP	Cartilage intermediate layer protein
COL1A1	Collagen type I, alpha 1
COL2A1	Collagen type II, alpha 1
COL10A1	Collagen type X, alpha 1
COMP	Cartilage oligomeric matrix protein
CRTAP	Cartilage associated protein
DCN	Decorin
DMMB	Dimethyl methylene blue
cDNA	Complementary deoxyribonucleic acid
ECM	Extracellular matrix
EDTA	Ethylenediaminetetraacetic acid
FBS	Fetal bovine serum
FC	Fold change
GAG	Glycosaminoglycan
GAPDH	Glyceraldehyde-3-phosphate dehydrogenase
gDNA	Genomic deoxyribonucleic acid
GDP	Gross domestic product
HPRT1	Hypoxanthine phosphoribosyltransferase 1
IBSP	Integrin-binding sialoprotein
ID1	Inhibitor of DNA Binding 1, HLH Protein
IL1β	Interleukin 1β
IVD	Intervertebral disc
MMP	Matrix metalloproteinases
NOG	Noggin
NP	Nucleus pulposus
POSTN	Periostin (osteoblast-specific factor)
RNA	Ribonucleic acid
RTD-PCR	Real-time detection polymerase chain reaction
RUNX2	RUNX family transcription factor 2
SEM	Standard error of the mean
SERPINE1	Serpin family E member 1
SMAD7	SMAD Family Member 7
SOX9	SRY (sex determining region Y)-box 9
SPP1	Secreted phosphoprotein 1 (osteopontin)
TGFβ1	Transforming growth factor β1
TNFα	Tumor necrosis factor α
