# Supplementary material for: Degeneration of Lumbar Intervertebral Discs: Characterization of Anulus Fibrosus Tissue and Cells of Different Degeneration Grades
Source: Int J Mol Sci. 2020 Mar 21;21(6):2165. doi: 10.3390/ijms21062165 (PMC7139657; doi:10.3390/ijms21062165)
Supplement: Supplementary file 1 [file ijms-21-02165-s001.zip › Supplemental Table 1.docx]

Table S1: Differentially regulated genes in native AF tissue of IVDs with mild and severe degeneration grades

| **Affymetrix ID** | **Symbol** | **Mean Signal Mild Degeneration** | **Mean Signal Severe Degeneration** | **Fold Change** | **Name** |
| --- | --- | --- | --- | --- | --- |
| 210397_at | DEFB1 | 1264.1 | 82.2 | 29.40 | defensin, beta 1 |
| 219837_s_at | CYTL1 | 22918.8 | 1259.4 | 15.28 | cytokine-like 1 |
| 205765_at | CYP3A5 | 228.5 | 20.4 | 12.51 | cytochrome P450, family 3, subfamily A, polypeptide 5 |
| 219667_s_at | BANK1 | 1680.9 | 185.1 | 12.41 | B-cell scaffold protein with ankyrin repeats 1 |
| 224367_at | BEX2 | 431.8 | 40.1 | 12.41 | brain expressed X-linked 2 |
| 241412_at | BTC | 108.3 | 16.7 | 9.70 | betacellulin |
| 219255_x_at | IL17RB | 171.5 | 23.3 | 9.62 | interleukin 17 receptor B |
| 203438_at | STC2 | 696.5 | 78.9 | 9.55 | stanniocalcin 2 |
| 210683_at | NRTN | 136.6 | 20.5 | 8.84 | neurturin |
| 200606_at | DSP | 2131.3 | 426.0 | 8.71 | desmoplakin |
| 239336_at | THBS1 | 71.5 | 8.7 | 8.44 | Thrombospondin 1 |
| 239461_at | GALNTL2 | 742.7 | 89.5 | 8.31 | UDP-N-acetyl-alpha-D-galactosamine:polypeptide N-acetylgalactosaminyltransferase-like 2 |
| 235737_at | TSLP | 137.8 | 16.0 | 8.31 | thymic stromal lymphopoietin |
| 204602_at | DKK1 | 3364.5 | 381.0 | 8.12 | dickkopf homolog 1 (Xenopus laevis) |
| 230087_at | PRIMA1 | 176.8 | 42.8 | 7.70 | proline rich membrane anchor 1 |
| 229158_at | WNK4 | 327.2 | 36.5 | 7.46 | WNK lysine deficient protein kinase 4 |
| 204223_at | PRELP | 34784.7 | 3895.4 | 7.35 | proline/arginine-rich end leucine-rich repeat protein |
| 220518_at | ABI3BP | 483.0 | 58.3 | 7.29 | ABI family, member 3 (NESH) binding protein |
| 228307_at | EMILIN3 | 636.6 | 69.4 | 7.02 | elastin microfibril interfacer 3 |
| 205728_at | ODZ1 | 200.8 | 35.0 | 7.02 | odz, odd Oz/ten-m homolog 1(Drosophila) |
| 227641_at | FBXL16 | 563.6 | 69.5 | 6.96 | F-box and leucine-rich repeat protein 16 |
| 1555958_at | CRTAC1 | 632.8 | 116.8 | 6.65 | cartilage acidic protein 1 |
| 219305_x_at | FBXO2 | 952.1 | 158.3 | 6.65 | F-box protein 2 |
| 203453_at | SCNN1A | 248.8 | 34.0 | 6.65 | sodium channel, nonvoltage-gated 1 alpha |
| 240187_at | PPP1R3C | 482.6 | 68.1 | 6.25 | protein phosphatase 1, regulatory (inhibitor) subunit 3C |
| 219615_s_at | KCNK5 | 522.0 | 103.7 | 6.20 | potassium channel, subfamily K, member 5 |
| 204776_at | THBS4 | 2048.3 | 651.0 | 5.97 | thrombospondin 4 |
| 214091_s_at | GPX3 | 11915.1 | 1856.7 | 5.79 | glutathione peroxidase 3 (plasma) |
| 206227_at | CILP | 40338.7 | 6699.4 | 5.74 | cartilage intermediate layer protein, nucleotide pyrophosphohydrolase |
| 233002_at | PPP4R4 | 288.1 | 61.0 | 5.66 | protein phosphatase 4, regulatory subunit 4 |
| 204154_at | CDO1 | 19483.6 | 3224.4 | 5.61 | cysteine dioxygenase, type I |
| 203305_at | F13A1 | 7380.1 | 1374.1 | 5.61 | coagulation factor XIII, A1 polypeptide |
| 237465_at | USP53 | 680.1 | 87.8 | 5.49 | ubiquitin specific peptidase 53 |
| 228758_at | BCL6 | 1925.8 | 483.4 | 5.36 | B-cell CLL/lymphoma 6 |
| 204724_s_at | COL9A3 | 2519.6 | 603.7 | 5.36 | collagen, type IX, alpha 3 |
| 204932_at | TNFRSF11B | 3132.7 | 833.0 | 5.36 | tumor necrosis factor receptor superfamily, member 11b |
| 206439_at | EPYC | 1115.3 | 440.9 | 5.32 | epiphycan |
| 210155_at | MYOC | 656.1 | 90.7 | 5.32 | myocilin, trabecular meshwork inducible glucocorticoid response |
| 237382_at | GLT25D2 | 182.6 | 26.7 | 5.28 | glycosyltransferase 25 domain containing 2 |
| 203961_at | NEBL | 2078.2 | 505.7 | 5.24 | nebulette |
| 225996_at | LONRF2 | 134.3 | 29.6 | 5.20 | LON peptidase N-terminal domain and ring finger 2 |
| 201690_s_at | TPD52 | 1013.5 | 323.7 | 5.16 | tumor protein D52 |
| 218899_s_at | BAALC | 653.0 | 154.4 | 5.12 | brain and acute leukemia, cytoplasmic |
| 211276_at | TCEAL2 | 1503.7 | 371.0 | 5.08 | transcription elongation factor A (SII)-like 2 |
| 226281_at | DNER | 1459.0 | 497.2 | 5.00 | delta/notch-like EGF repeat containing |
| 204754_at | HLF | 518.3 | 152.1 | 5.00 | hepatic leukemia factor |
| 206486_at | LAG3 | 524.5 | 109.5 | 5.00 | lymphocyte-activation gene 3 |
| 227198_at | AFF3 | 1487.6 | 313.9 | 4.96 | AF4/FMR2 family, member 3 |
| 219295_s_at | PCOLCE2 | 18321.3 | 3608.9 | 4.96 | procollagen C-endopeptidase enhancer 2 |
| 238513_at | PRRG4 | 293.3 | 62.7 | 4.96 | Proline rich Gla (G-carboxyglutamic acid) 4 (transmembrane) |
| 218237_s_at | SLC38A1 | 695.9 | 151.0 | 4.92 | solute carrier family 38, member 1 |
| 223836_at | FGFBP2 | 28248.8 | 6316.7 | 4.74 | fibroblast growth factor binding protein 2 |
| 205833_s_at | PART1 | 438.1 | 92.6 | 4.74 | prostate androgen-regulated transcript 1 |
| 211751_at | PDE4DIP | 122.6 | 18.1 | 4.67 | phosphodiesterase 4D interacting protein |
| 229151_at | SLC14A1 | 1420.2 | 253.5 | 4.59 | solute carrier family 14 (urea transporter), member 1 (Kidd blood group) |
| 212565_at | STK38L | 2303.9 | 610.1 | 4.56 | serine/threonine kinase 38 like |
| 229963_at | BEX5 | 528.4 | 114.7 | 4.52 | brain expressed, X-linked 5 |
| 240556_at | DCN | 367.8 | 82.6 | 4.52 | Decorin |
| 206560_s_at | MIA | 4628.5 | 1140.2 | 4.52 | melanoma inhibitory activity |
| 229170_s_at | TTC18 | 229.0 | 40.4 | 4.52 | tetratricopeptide repeat domain 18 |
| 204422_s_at | FGF2 | 1551.2 | 517.2 | 4.49 | fibroblast growth factor 2 (basic) |
| 235077_at | MEG3 | 217.8 | 69.2 | 4.42 | maternally expressed 3 (non-protein coding) |
| 203662_s_at | TMOD1 | 696.2 | 166.6 | 4.42 | tropomodulin 1 |
| 229764_at | TPRG1 | 472.7 | 81.2 | 4.42 | tumor protein p63 regulated 1 |
| 227401_at | IL17D | 738.7 | 170.1 | 4.39 | interleukin 17D |
| 211429_s_at | SERPINA1 | 7944.1 | 2144.0 | 4.39 | serpin peptidase inhibitor, clade A (alpha-1 antiproteinase, antitrypsin), member 1 |
| 205679_x_at | ACAN | 6901.3 | 2063.6 | 4.32 | aggrecan |
| 205616_at | KCNA6 | 288.4 | 72.2 | 4.29 | potassium voltage-gated channel, shaker-related subfamily, member 6 |
| 1552289_a_at | CILP2 | 6823.0 | 1761.8 | 4.25 | cartilage intermediate layer protein 2 |
| 203697_at | FRZB | 3293.0 | 585.8 | 4.22 | frizzled-related protein |
| 227059_at | GPC6 | 3743.3 | 1035.5 | 4.22 | glypican 6 |
| 220283_at | HHIPL2 | 1075.4 | 338.8 | 4.19 | HHIP-like 2 |
| 209398_at | HIST1H1C | 633.3 | 129.6 | 4.19 | histone cluster 1, H1c |
| 229310_at | KLHL29 | 1874.8 | 476.1 | 4.19 | kelch-like 29 (Drosophila) |
| 205236_x_at | SOD3 | 1529.0 | 362.9 | 4.16 | superoxide dismutase 3, extracellular |
| 206448_at | ZNF365 | 441.0 | 99.7 | 4.13 | zinc finger protein 365 |
| 215043_s_at | SMA4 /// SMA5 | 98.0 | 29.7 | 4.09 | glucuronidase, beta pseudogene /// glucuronidase, beta pseudogene |
| 232054_at | PCDH20 | 201.0 | 71.2 | 4.06 | protocadherin 20 |
| 218180_s_at | EPS8L2 | 391.2 | 96.5 | 4.03 | EPS8-like 2 |
| 212810_s_at | SLC1A4 | 1252.4 | 354.0 | 3.91 | solute carrier family 1 (glutamate/neutral amino acid transporter), member 4 |
| 205366_s_at | HOXB6 | 704.3 | 202.6 | 3.88 | homeobox B6 |
| 229667_s_at | HOXB8 | 397.4 | 103.8 | 3.88 | homeobox B8 |
| 226179_at | SLC25A37 | 2908.6 | 812.5 | 3.88 | solute carrier family 25, member 37 |
| 204022_at | WWP2 | 4962.4 | 1252.0 | 3.88 | WW domain containing E3 ubiquitin protein ligase 2 |
| 227240_at | NGEF | 1106.8 | 344.1 | 3.82 | neuronal guanine nucleotide exchange factor |
| 209686_at | S100B | 1099.3 | 321.9 | 3.82 | S100 calcium binding protein B |
| 226424_at | CAPS | 1538.3 | 355.8 | 3.79 | calcyphosine |
| 208433_s_at | LRP8 | 294.9 | 98.2 | 3.79 | low density lipoprotein receptor-related protein 8, apolipoprotein e receptor |
| 206869_at | CHAD | 4939.4 | 1664.2 | 3.76 | chondroadherin |
| 214401_at | PAX1 | 1471.5 | 408.5 | 3.76 | paired box 1 |
| 231735_s_at | MALAT1 | 756.2 | 150.3 | 3.73 | metastasis associated lung adenocarcinoma transcript 1 (non-protein coding) |
| 205330_at | MN1 | 2159.1 | 610.2 | 3.73 | meningioma (disrupted in balanced translocation) 1 |
| 205475_at | SCRG1 | 16195.5 | 4919.9 | 3.70 | stimulator of chondrogenesis 1 |
| 226325_at | ADSSL1 | 617.7 | 219.0 | 3.68 | adenylosuccinate synthase like 1 |
| 229228_at | CREB5 | 863.8 | 283.7 | 3.68 | cAMP responsive element binding protein 5 |
| 223690_at | LTBP2 | 4206.8 | 1284.0 | 3.68 | latent transforming growth factor beta binding protein 2 |
| 207016_s_at | ALDH1A2 | 979.1 | 238.8 | 3.65 | aldehyde dehydrogenase 1 family, member A2 |
| 209094_at | DDAH1 | 4101.8 | 1198.1 | 3.65 | dimethylarginine dimethylaminohydrolase 1 |
| 205334_at | S100A1 | 524.0 | 142.1 | 3.65 | S100 calcium binding protein A1 |
| 226344_at | ZMAT1 | 452.8 | 113.2 | 3.62 | zinc finger, matrin type 1 |
| 231164_at | ABCA17P | 158.7 | 33.1 | 3.59 | ATP-binding cassette, sub-family A (ABC1), member 17 (pseudogene) |
| 210605_s_at | MFGE8 | 2626.8 | 702.1 | 3.56 | milk fat globule-EGF factor 8 protein |
| 239726_at | ANK3 | 839.5 | 198.0 | 3.51 | Ankyrin 3, node of Ranvier (ankyrin G) |
| 225285_at | BCAT1 | 10865.7 | 2627.7 | 3.51 | branched chain aminotransferase 1, cytosolic |
| 205713_s_at | COMP | 40537.4 | 11198.6 | 3.46 | cartilage oligomeric matrix protein |
| 205466_s_at | HS3ST1 | 698.1 | 225.6 | 3.46 | heparan sulfate (glucosamine) 3-O-sulfotransferase 1 |
| 201362_at | IVNS1ABP | 6153.5 | 1927.8 | 3.46 | influenza virus NS1A binding protein |
| 232180_at | UGP2 | 429.4 | 125.9 | 3.46 | UDP-glucose pyrophosphorylase 2 |
| 235496_at | HRCT1 | 584.0 | 167.3 | 3.43 | histidine rich carboxyl terminus 1 |
| 229032_at | WSCD2 | 218.2 | 94.8 | 3.40 | WSC domain containing 2 |
| 209283_at | CRYAB | 6623.2 | 2091.2 | 3.38 | crystallin, alpha B |
| 203851_at | IGFBP6 | 2400.3 | 614.6 | 3.32 | insulin-like growth factor binding protein 6 |
| 211596_s_at | LRIG1 | 883.7 | 356.5 | 3.32 | leucine-rich repeats and immunoglobulin-like domains 1 |
| 228255_at | ALS2CR4 | 320.2 | 105.2 | 3.27 | amyotrophic lateral sclerosis 2 (juvenile) chromosome region, candidate 4 |
| 227145_at | LOXL4 | 1051.5 | 245.1 | 3.27 | lysyl oxidase-like 4 |
| 206941_x_at | SEMA3E | 496.3 | 148.8 | 3.27 | sema domain, immunoglobulin domain (Ig), short basic domain, secreted, (semaphorin) 3E |
| 203685_at | BCL2 | 1014.7 | 322.9 | 3.22 | B-cell CLL/lymphoma 2 |
| 213622_at | COL9A2 | 1322.3 | 470.2 | 3.22 | collagen, type IX, alpha 2 |
| 225871_at | STEAP2 | 891.0 | 360.4 | 3.22 | six transmembrane epithelial antigen of the prostate 2 |
| 228414_at | KCNMA1 | 229.2 | 66.0 | 3.20 | potassium large conductance calcium-activated channel, subfamily M, alpha member 1 |
| 227654_at | FAM65C | 229.9 | 60.2 | 3.17 | family with sequence similarity 65, member C |
| 229674_at | SERTAD4 | 1791.9 | 505.4 | 3.17 | SERTA domain containing 4 |
| 202709_at | FMOD | 24907.0 | 7901.3 | 3.15 | fibromodulin |
| 225407_at | MBP | 443.4 | 193.7 | 3.15 | myelin basic protein |
| 205499_at | SRPX2 | 2614.8 | 1002.2 | 3.13 | sushi-repeat-containing protein, X-linked 2 |
| 232099_at | PCDHB16 | 632.3 | 218.9 | 3.08 | protocadherin beta 16 |
| 219093_at | PID1 | 1938.1 | 828.9 | 3.08 | phosphotyrosine interaction domain containing 1 |
| 1552365_at | SCIN | 2275.6 | 858.0 | 3.08 | scinderin |
| 218692_at | GOLSYN | 1246.2 | 450.5 | 3.05 | Golgi-localized protein |
| 238803_at | HECTD2 | 416.7 | 120.0 | 3.05 | HECT domain containing 2 |
| 229797_at | MCOLN3 | 668.0 | 209.3 | 3.01 | mucolipin 3 |
| 229657_at | THRB | 477.3 | 154.4 | 2.99 | thyroid hormone receptor, beta (erythroblastic leukemia viral (v-erb-a) oncogene homolog 2, avian) |
| 213217_at | ADCY2 | 726.8 | 215.3 | 2.96 | adenylate cyclase 2 (brain) |
| 204224_s_at | GCH1 | 1634.8 | 510.4 | 2.92 | GTP cyclohydrolase 1 |
| 238481_at | MGP | 505.1 | 202.7 | 2.92 | matrix Gla protein |
| 238778_at | MPP7 | 290.4 | 99.2 | 2.92 | membrane protein, palmitoylated 7 (MAGUK p55 subfamily member 7) |
| 224973_at | FAM46A | 2601.8 | 798.3 | 2.89 | family with sequence similarity 46, member A |
| 229281_at | NPAS3 | 223.4 | 83.6 | 2.89 | neuronal PAS domain protein 3 |
| 222722_at | OGN | 18848.1 | 6196.3 | 2.89 | osteoglycin |
| 224325_at | FZD8 | 2196.8 | 634.4 | 2.87 | frizzled homolog 8 (Drosophila) |
| 226003_at | KIF21A | 462.4 | 188.3 | 2.85 | kinesin family member 21A |
| 227379_at | MBOAT1 | 930.7 | 320.3 | 2.85 | membrane bound O-acyltransferase domain containing 1 |
| 223842_s_at | SCARA3 | 996.8 | 374.6 | 2.83 | scavenger receptor class A, member 3 |
| 205141_at | ANG | 476.3 | 196.0 | 2.76 | angiogenin, ribonuclease, RNase A family, 5 |
| 206170_at | ADRB2 | 874.3 | 270.7 | 2.72 | adrenergic, beta-2-, receptor, surface |
| 225915_at | CAB39L | 614.0 | 257.9 | 2.72 | calcium binding protein 39-like |
| 202936_s_at | SOX9 | 3477.8 | 1730.6 | 2.72 | SRY (sex determining region Y)-box 9 |
| 235749_at | UGGT2 | 360.5 | 134.8 | 2.72 | UDP-glucose glycoprotein glucosyltransferase 2 |
| 208792_s_at | CLU | 41576.6 | 13371.9 | 2.70 | clusterin |
| 226769_at | FIBIN | 5498.7 | 1858.1 | 2.70 | fin bud initiation factor homolog (zebrafish) |
| 213353_at | ABCA5 | 824.3 | 303.3 | 2.68 | ATP-binding cassette, sub-family A (ABC1), member 5 |
| 213974_at | ADAMTSL3 | 500.8 | 204.7 | 2.66 | ADAMTS-like 3 |
| 223786_at | CHST6 | 718.2 | 239.2 | 2.66 | carbohydrate (N-acetylglucosamine 6-O) sulfotransferase 6 |
| 203678_at | MTMR15 | 732.5 | 285.2 | 2.66 | myotubularin related protein 15 |
| 229141_at | WDR33 | 252.0 | 80.7 | 2.66 | WD repeat domain 33 |
| 201540_at | FHL1 | 18325.6 | 6274.4 | 2.64 | four and a half LIM domains 1 |
| 219949_at | LRRC2 | 279.5 | 116.4 | 2.64 | leucine rich repeat containing 2 |
| 207076_s_at | ASS1 | 3871.8 | 1353.0 | 2.62 | argininosuccinate synthetase 1 |
| 1552935_at | ZCCHC5 | 290.3 | 98.2 | 2.60 | zinc finger, CCHC domain containing 5 |
| 204793_at | GPRASP1 | 845.5 | 381.1 | 2.58 | G protein-coupled receptor associated sorting protein 1 |
| 225150_s_at | RTKN | 219.3 | 75.7 | 2.58 | rhotekin |
| 204326_x_at | MT1X | 14319.0 | 5999.3 | 2.56 | metallothionein 1X |
| 202022_at | ALDOC | 658.5 | 283.7 | 2.54 | aldolase C, fructose-bisphosphate |
| 227486_at | NT5E | 541.6 | 214.0 | 2.54 | 5'-nucleotidase, ecto (CD73) |
| 226926_at | DMKN | 248.3 | 85.2 | 2.52 | dermokine |
| 202481_at | DHRS3 | 697.7 | 266.6 | 2.46 | dehydrogenase/reductase (SDR family) member 3 |
| 205066_s_at | ENPP1 | 1351.1 | 563.8 | 2.46 | ectonucleotide pyrophosphatase/phosphodiesterase 1 |
| 214395_x_at | EEF1D | 260.5 | 103.6 | 2.44 | eukaryotic translation elongation factor 1 delta (guanine nucleotide exchange protein) |
| 202748_at | GBP2 | 1613.5 | 537.1 | 2.44 | guanylate binding protein 2, interferon-inducible |
| 204396_s_at | GRK5 | 1165.5 | 482.6 | 2.42 | G protein-coupled receptor kinase 5 |
| 206766_at | ITGA10 | 4617.5 | 1861.6 | 2.42 | integrin, alpha 10 |
| 33323_r_at | SFN | 310.3 | 135.7 | 2.42 | stratifin |
| 200650_s_at | LDHA | 15443.1 | 5990.7 | 2.41 | lactate dehydrogenase A |
| 212991_at | FBXO9 | 207.6 | 78.3 | 2.39 | F-box protein 9 |
| 205074_at | SLC22A5 | 217.5 | 103.6 | 2.39 | solute carrier family 22 (organic cation/carnitine transporter), member 5 |
| 1568868_at | CYP27C1 | 453.6 | 184.9 | 2.37 | cytochrome P450, family 27, subfamily C, polypeptide 1 |
| 201289_at | CYR61 | 10725.5 | 5011.9 | 2.37 | cysteine-rich, angiogenic inducer, 61 |
| 1552735_at | PCDHGA4 | 153.1 | 66.3 | 2.37 | protocadherin gamma subfamily A, 4 |
| 216333_x_at | TNXA /// TNXB | 1854.5 | 863.4 | 2.37 | tenascin XA pseudogene /// tenascin XB |
| 219087_at | ASPN | 20240.5 | 8186.0 | 2.35 | asporin |
| 219064_at | ITIH5 | 926.4 | 433.2 | 2.35 | inter-alpha (globulin) inhibitor H5 |
| 225627_s_at | CACHD1 | 948.5 | 434.8 | 2.32 | cache domain containing 1 |
| 1553972_a_at | CBS | 239.0 | 132.4 | 2.32 | cystathionine-beta-synthase |
| 227308_x_at | LTBP3 | 735.4 | 341.9 | 2.32 | latent transforming growth factor beta binding protein 3 |
| 202289_s_at | TACC2 | 788.2 | 336.5 | 2.32 | transforming, acidic coiled-coil containing protein 2 |
| 228088_at | SESTD1 | 469.0 | 223.6 | 2.30 | SEC14 and spectrin domains 1 |
| 214329_x_at | TNFSF10 | 1162.3 | 585.0 | 2.30 | tumor necrosis factor (ligand) superfamily, member 10 |
| 213629_x_at | MT1F | 3338.5 | 1189.2 | 2.28 | metallothionein 1F |
| 224565_at | NEAT1 | 6627.5 | 3208.1 | 2.28 | nuclear paraspeckle assembly transcript 1 (non-protein coding) |
| 212179_at | SFRS18 | 1117.4 | 470.5 | 2.28 | splicing factor, arginine/serine-rich 18 |
| 213800_at | CFH | 3704.5 | 1502.7 | 2.26 | complement factor H |
| 213645_at | ENOSF1 | 628.4 | 232.8 | 2.26 | enolase superfamily member 1 |
| 213492_at | COL2A1 | 23565.9 | 9555.6 | 2.19 | collagen, type II, alpha 1 |
| 234192_s_at | GKAP1 | 232.6 | 95.0 | 2.19 | G kinase anchoring protein 1 |
| 208131_s_at | PTGIS | 1867.9 | 1019.5 | 2.19 | prostaglandin I2 (prostacyclin) synthase |
| 221569_at | AHI1 | 890.1 | 389.0 | 2.16 | Abelson helper integration site 1 |
| 226656_at | CRTAP | 1000.4 | 446.4 | 2.16 | cartilage associated protein |
| 212345_s_at | CREB3L2 | 3020.9 | 1619.1 | 2.14 | cAMP responsive element binding protein 3-like 2 |
| 225520_at | MTHFD1L | 840.5 | 410.7 | 2.14 | methylenetetrahydrofolate dehydrogenase (NADP+ dependent) 1-like |
| 206176_at | BMP6 | 403.8 | 229.7 | 2.13 | bone morphogenetic protein 6 |
| 202259_s_at | N4BP2L2 | 708.5 | 337.9 | 2.13 | NEDD4 binding protein 2-like 2 |
| 201581_at | TMX4 | 2389.1 | 1055.2 | 2.13 | thioredoxin-related transmembrane protein 4 |
| 204457_s_at | GAS1 | 8859.1 | 4297.5 | 2.09 | growth arrest-specific 1 |
| 1552733_at | KLHDC1 | 598.2 | 254.2 | 2.09 | kelch domain containing 1 |
| 233952_s_at | ZNF295 | 857.8 | 420.6 | 2.09 | zinc finger protein 295 |
| 214151_s_at | CCPG1 | 1508.4 | 889.3 | 2.08 | cell cycle progression 1 |
| 228370_at | SNRPN | 700.8 | 297.9 | 2.08 | small nuclear ribonucleoprotein polypeptide N |
| 213737_x_at | GOLGA8C /// GOLGA8D /// GOLGA8E /// GOLGA8G /// LOC653061 | 3949.2 | 1778.3 | 2.06 | golgi autoantigen, golgin subfamily a, 8C /// golgi autoantigen, golgin subfamily a, 8D /// golgi autoantigen, golgin subfamily a, 8E /// golgi autoantigen, golgin subfamily a, 8G /// similar to Golgin subfamily A member 8-like protein 1 |
| 218974_at | SOBP | 1647.0 | 723.2 | 2.06 | sine oculis binding protein homolog (Drosophila) |
| 202723_s_at | FOXO1 | 520.0 | 265.6 | 2.03 | forkhead box O1 |
| 234981_x_at | CMBL | 3303.6 | 1794.2 | 2.02 | carboxymethylenebutenolidase homolog (Pseudomonas) |
| 201592_at | EIF3H | 6304.8 | 2888.7 | 2.02 | eukaryotic translation initiation factor 3, subunit H |
| 221897_at | TRIM52 | 504.0 | 252.5 | 2.00 | tripartite motif-containing 52 |
| 220199_s_at | AIDA | 404.5 | 851.3 | -2.00 | axin interactor, dorsalization associated |
| 208074_s_at | AP2S1 | 381.1 | 808.8 | -2.00 | adaptor-related protein complex 2, sigma 1 subunit |
| 211559_s_at | CCNG2 | 89.0 | 187.9 | -2.00 | cyclin G2 |
| 209158_s_at | CYTH2 | 169.4 | 304.8 | -2.00 | cytohesin 2 |
| 35666_at | SEMA3F | 207.3 | 615.4 | -2.00 | sema domain, immunoglobulin domain (Ig), short basic domain, secreted, (semaphorin) 3F |
| 217766_s_at | TMEM50A | 291.5 | 632.6 | -2.00 | transmembrane protein 50A |
| 200611_s_at | WDR1 | 444.6 | 940.6 | -2.00 | WD repeat domain 1 |
| 1554980_a_at | ATF3 | 102.5 | 179.9 | -2.02 | activating transcription factor 3 |
| 208867_s_at | CSNK1A1 | 247.7 | 452.2 | -2.02 | casein kinase 1, alpha 1 |
| 227370_at | FAM171B | 337.3 | 571.6 | -2.02 | family with sequence similarity 171, member B |
| 212335_at | GNS | 342.0 | 766.2 | -2.02 | glucosamine (N-acetyl)-6-sulfatase |
| 243296_at | NAMPT | 451.2 | 1074.5 | -2.03 | Nicotinamide phosphoribosyltransferase |
| 224901_at | SCD5 | 249.4 | 432.4 | -2.03 | stearoyl-CoA desaturase 5 |
| 201194_at | SEPW1 | 902.5 | 1842.8 | -2.03 | selenoprotein W, 1 |
| 216899_s_at | SKAP2 | 113.8 | 222.4 | -2.03 | src kinase associated phosphoprotein 2 |
| 218368_s_at | TNFRSF12A | 155.7 | 412.9 | -2.03 | tumor necrosis factor receptor superfamily, member 12A |
| 209732_at | CLEC2B | 562.8 | 1153.9 | -2.05 | C-type lectin domain family 2, member B |
| 222473_s_at | ERBB2IP | 278.1 | 481.7 | -2.05 | erbb2 interacting protein |
| 228418_at | EXOC5 | 101.0 | 211.2 | -2.05 | exocyst complex component 5 |
| 216693_x_at | HDGFRP3 | 293.5 | 609.0 | -2.05 | hepatoma-derived growth factor, related protein 3 |
| 224247_s_at | MRPS10 | 70.3 | 155.6 | -2.05 | mitochondrial ribosomal protein S10 |
| 203072_at | MYO1E | 74.0 | 196.6 | -2.05 | myosin IE |
| 202783_at | NNT | 118.2 | 313.5 | -2.05 | nicotinamide nucleotide transhydrogenase |
| 203238_s_at | NOTCH3 | 229.5 | 499.0 | -2.05 | Notch homolog 3 (Drosophila) |
| 210875_s_at | ZEB1 | 201.4 | 439.0 | -2.05 | zinc finger E-box binding homeobox 1 |
| 201954_at | ARPC1B | 413.6 | 1197.1 | -2.06 | actin related protein 2/3 complex, subunit 1B, 41kDa |
| 242439_s_at | ASXL1 | 138.0 | 268.4 | -2.06 | additional sex combs like 1 (Drosophila) |
| 232204_at | EBF1 | 300.1 | 686.4 | -2.06 | early B-cell factor 1 |
| 215017_s_at | FNBP1L | 293.9 | 527.0 | -2.06 | formin binding protein 1-like |
| 210830_s_at | PON2 | 302.7 | 543.5 | -2.06 | paraoxonase 2 |
| 203038_at | PTPRK | 561.1 | 1185.8 | -2.06 | protein tyrosine phosphatase, receptor type, K |
| 209882_at | RIT1 | 138.8 | 323.1 | -2.06 | Ras-like without CAAX 1 |
| 202241_at | TRIB1 | 699.6 | 1527.8 | -2.06 | tribbles homolog 1 (Drosophila) |
| 203052_at | C2 | 104.4 | 198.0 | -2.08 | complement component 2 |
| 226568_at | FAM102B | 423.4 | 814.0 | -2.08 | family with sequence similarity 102, member B |
| 225604_s_at | GLIPR2 | 82.5 | 172.1 | -2.08 | GLI pathogenesis-related 2 |
| 205991_s_at | PRRX1 | 262.5 | 502.3 | -2.08 | paired related homeobox 1 |
| 216591_s_at | SDHC | 112.0 | 237.5 | -2.08 | succinate dehydrogenase complex, subunit C, integral membrane protein, 15kDa |
| 226825_s_at | TMEM165 | 460.6 | 1004.1 | -2.08 | transmembrane protein 165 |
| 204140_at | TPST1 | 275.1 | 601.3 | -2.08 | tyrosylprotein sulfotransferase 1 |
| 211672_s_at | ARPC4 /// TTLL3 | 181.6 | 348.3 | -2.09 | actin related protein 2/3 complex, subunit 4, 20kDa /// tubulin tyrosine ligase-like family, member 3 |
| 226847_at | FST | 74.7 | 148.4 | -2.09 | follistatin |
| 205729_at | OSMR | 108.7 | 237.2 | -2.09 | oncostatin M receptor |
| 200604_s_at | PRKAR1A | 135.8 | 272.3 | -2.09 | protein kinase, cAMP-dependent, regulatory, type I, alpha (tissue specific extinguisher 1) |
| 204279_at | PSMB9 | 223.2 | 492.9 | -2.09 | proteasome (prosome, macropain) subunit, beta type, 9 (large multifunctional peptidase 2) |
| 214853_s_at | SHC1 | 1439.8 | 3158.0 | -2.09 | SHC (Src homology 2 domain containing) transforming protein 1 |
| 225606_at | BCL2L11 | 180.6 | 378.6 | -2.11 | BCL2-like 11 (apoptosis facilitator) |
| 202701_at | BMP1 | 123.0 | 285.6 | -2.11 | bone morphogenetic protein 1 |
| 201179_s_at | GNAI3 | 175.4 | 338.0 | -2.11 | guanine nucleotide binding protein (G protein), alpha inhibiting activity polypeptide 3 |
| 200736_s_at | GPX1 | 1348.8 | 3212.5 | -2.11 | glutathione peroxidase 1 |
| 209179_s_at | MBOAT7 | 124.2 | 234.5 | -2.11 | membrane bound O-acyltransferase domain containing 7 |
| 226035_at | USP31 | 171.1 | 310.5 | -2.11 | ubiquitin specific peptidase 31 |
| 1554417_s_at | APH1A | 106.5 | 219.8 | -2.13 | anterior pharynx defective 1 homolog A (C. elegans) |
| 1555419_a_at | ASAH1 | 419.0 | 849.4 | -2.13 | N-acylsphingosine amidohydrolase (acid ceramidase) 1 |
| 204170_s_at | CKS2 | 132.0 | 362.9 | -2.13 | CDC28 protein kinase regulatory subunit 2 |
| 1554411_at | CTNNB1 | 700.9 | 1380.0 | -2.13 | catenin (cadherin-associated protein), beta 1, 88kDa |
| 227961_at | CTSB | 485.0 | 1191.2 | -2.13 | cathepsin B |
| 226876_at | FAM101B | 405.7 | 809.8 | -2.13 | family with sequence similarity 101, member B |
| 210338_s_at | HSPA8 | 2439.6 | 5129.6 | -2.13 | heat shock 70kDa protein 8 |
| 202626_s_at | LYN | 72.8 | 172.1 | -2.13 | v-yes-1 Yamaguchi sarcoma viral related oncogene homolog |
| 208296_x_at | TNFAIP8 | 111.8 | 235.3 | -2.13 | tumor necrosis factor, alpha-induced protein 8 |
| 201883_s_at | B4GALT1 | 212.9 | 417.2 | -2.14 | UDP-Gal:betaGlcNAc beta 1,4- galactosyltransferase, polypeptide 1 |
| 215794_x_at | GLUD2 | 194.6 | 400.7 | -2.14 | glutamate dehydrogenase 2 |
| 228950_s_at | GPR177 | 139.4 | 271.0 | -2.14 | G protein-coupled receptor 177 |
| 217599_s_at | MDFIC | 61.6 | 148.2 | -2.14 | MyoD family inhibitor domain containing |
| 209481_at | SNRK | 586.9 | 1171.6 | -2.14 | SNF related kinase |
| 1567107_s_at | TPM4 | 135.7 | 337.9 | -2.14 | tropomyosin 4 |
| 204950_at | CARD8 | 107.3 | 224.0 | -2.16 | caspase recruitment domain family, member 8 |
| 211922_s_at | CAT | 146.8 | 333.4 | -2.16 | catalase |
| 202411_at | IFI27 | 524.8 | 1216.3 | -2.16 | interferon, alpha-inducible protein 27 |
| 35147_at | MCF2L | 144.1 | 325.0 | -2.16 | MCF.2 cell line derived transforming sequence-like |
| 205934_at | PLCL1 | 77.0 | 154.3 | -2.16 | phospholipase C-like 1 |
| 205801_s_at | RASGRP3 | 114.6 | 236.2 | -2.16 | RAS guanyl releasing protein 3 (calcium and DAG-regulated) |
| 201207_at | TNFAIP1 | 272.5 | 575.0 | -2.16 | tumor necrosis factor, alpha-induced protein 1 (endothelial) |
| 202079_s_at | TRAK1 | 62.8 | 129.4 | -2.16 | trafficking protein, kinesin binding 1 |
| 209846_s_at | BTN3A2 | 107.8 | 246.6 | -2.18 | butyrophilin, subfamily 3, member A2 |
| 206756_at | CHST7 | 82.3 | 204.5 | -2.18 | carbohydrate (N-acetylglucosamine 6-O) sulfotransferase 7 |
| 235177_at | FAM119A | 68.7 | 142.4 | -2.18 | family with sequence similarity 119, member A |
| 1553764_a_at | JUB | 52.8 | 155.6 | -2.18 | jub, ajuba homolog (Xenopus laevis) |
| 242794_at | MAML3 | 72.0 | 165.7 | -2.18 | mastermind-like 3 (Drosophila) |
| 204518_s_at | PPIC | 547.5 | 1219.0 | -2.18 | peptidylprolyl isomerase C (cyclophilin C) |
| 202381_at | ADAM9 | 737.0 | 1561.5 | -2.19 | ADAM metallopeptidase domain 9 (meltrin gamma) |
| 202874_s_at | ATP6V1C1 | 225.2 | 484.0 | -2.19 | ATPase, H+ transporting, lysosomal 42kDa, V1 subunit C1 |
| 209298_s_at | ITSN1 | 142.3 | 327.5 | -2.19 | intersectin 1 (SH3 domain protein) |
| 208926_at | NEU1 | 132.9 | 313.3 | -2.19 | sialidase 1 (lysosomal sialidase) |
| 222846_at | RAB8B | 70.1 | 164.6 | -2.19 | RAB8B, member RAS oncogene family |
| 226576_at | ARHGAP26 | 76.6 | 153.0 | -2.21 | Rho GTPase activating protein 26 |
| 209788_s_at | ERAP1 | 182.9 | 397.3 | -2.21 | endoplasmic reticulum aminopeptidase 1 |
| 223386_at | FAM118B | 70.4 | 162.8 | -2.21 | family with sequence similarity 118, member B |
| 213918_s_at | NIPBL | 204.7 | 482.9 | -2.21 | Nipped-B homolog (Drosophila) |
| 225929_s_at | RNF213 | 131.5 | 331.5 | -2.21 | ring finger protein 213 |
| 201307_at | SEPT11 | 757.1 | 1432.5 | -2.21 | septin 11 |
| 225895_at | SYNPO2 | 225.0 | 500.1 | -2.21 | synaptopodin 2 |
| 209156_s_at | COL6A2 | 611.6 | 1412.3 | -2.23 | collagen, type VI, alpha 2 |
| 201579_at | FAT1 | 754.8 | 1709.9 | -2.23 | FAT tumor suppressor homolog 1 (Drosophila) |
| 207198_s_at | LIMS1 | 163.6 | 429.6 | -2.23 | LIM and senescent cell antigen-like domains 1 |
| 203823_at | RGS3 | 879.9 | 1803.7 | -2.23 | regulator of G-protein signaling 3 |
| 222154_s_at | SPATS2L | 939.3 | 2055.9 | -2.23 | spermatogenesis associated, serine-rich 2-like |
| 222557_at | STMN3 | 109.0 | 266.8 | -2.23 | stathmin-like 3 |
| 219423_x_at | TNFRSF25 | 79.2 | 212.7 | -2.23 | tumor necrosis factor receptor superfamily, member 25 |
| 212041_at | ATP6V0D1 | 319.5 | 703.8 | -2.24 | ATPase, H+ transporting, lysosomal 38kDa, V0 subunit d1 |
| 201950_x_at | CAPZB | 314.5 | 664.0 | -2.24 | capping protein (actin filament) muscle Z-line, beta |
| 213923_at | RAP2B | 524.6 | 1055.7 | -2.24 | RAP2B, member of RAS oncogene family |
| 219204_s_at | SRR | 55.6 | 111.0 | -2.24 | serine racemase |
| 206283_s_at | TAL1 | 115.3 | 249.9 | -2.24 | T-cell acute lymphocytic leukemia 1 |
| 1555797_a_at | ARPC5 | 250.7 | 582.0 | -2.26 | actin related protein 2/3 complex, subunit 5, 16kDa |
| 208712_at | CCND1 | 345.1 | 712.4 | -2.26 | cyclin D1 |
| 205565_s_at | FXN | 59.2 | 132.3 | -2.26 | frataxin |
| 204112_s_at | HNMT | 451.2 | 1017.0 | -2.26 | histamine N-methyltransferase |
| 223322_at | RASSF5 | 84.8 | 153.5 | -2.26 | Ras association (RalGDS/AF-6) domain family member 5 |
| 201663_s_at | SMC4 | 108.6 | 225.7 | -2.26 | structural maintenance of chromosomes 4 |
| 209751_s_at | TRAPPC2 /// TRAPPC2P1 | 144.7 | 319.1 | -2.26 | trafficking protein particle complex 2 /// trafficking protein particle complex 2 pseudogene 1 |
| 201946_s_at | CCT2 | 164.5 | 443.7 | -2.28 | chaperonin containing TCP1, subunit 2 (beta) |
| 1554679_a_at | LAPTM4B | 226.4 | 531.0 | -2.28 | lysosomal protein transmembrane 4 beta |
| 221261_x_at | MAGED4 /// MAGED4B | 96.2 | 224.4 | -2.28 | melanoma antigen family D, 4 /// melanoma antigen family D, 4B |
| 202607_at | NDST1 | 55.7 | 150.3 | -2.28 | N-deacetylase/N-sulfotransferase (heparan glucosaminyl) 1 |
| 212930_at | ATP2B1 | 130.3 | 246.9 | -2.30 | ATPase, Ca++ transporting, plasma membrane 1 |
| 226350_at | CHML | 135.2 | 321.4 | -2.30 | choroideremia-like (Rab escort protein 2) |
| 223047_at | CMTM6 | 318.5 | 824.3 | -2.30 | CKLF-like MARVEL transmembrane domain containing 6 |
| 222250_s_at | INTS7 | 66.3 | 114.6 | -2.30 | integrator complex subunit 7 |
| 202998_s_at | LOXL2 | 356.1 | 915.0 | -2.30 | lysyl oxidase-like 2 |
| 206584_at | LY96 | 214.9 | 614.9 | -2.30 | lymphocyte antigen 96 |
| 221002_s_at | TSPAN14 | 230.7 | 416.4 | -2.30 | tetraspanin 14 |
| 212940_at | COL6A1 | 435.1 | 1096.8 | -2.32 | collagen, type VI, alpha 1 |
| 215177_s_at | ITGA6 | 191.8 | 391.5 | -2.32 | integrin, alpha 6 |
| 200907_s_at | PALLD | 707.5 | 1722.3 | -2.32 | palladin, cytoskeletal associated protein |
| 205329_s_at | SNX4 | 74.5 | 184.0 | -2.32 | sorting nexin 4 |
| 204163_at | EMILIN1 | 159.7 | 357.0 | -2.33 | elastin microfibril interfacer 1 |
| 206335_at | GALNS | 253.2 | 562.7 | -2.33 | galactosamine (N-acetyl)-6-sulfate sulfatase |
| 221942_s_at | GUCY1A3 | 284.8 | 630.9 | -2.33 | guanylate cyclase 1, soluble, alpha 3 |
| 201818_at | LPCAT1 | 218.6 | 543.3 | -2.33 | lysophosphatidylcholine acyltransferase 1 |
| 205463_s_at | PDGFA | 219.9 | 448.3 | -2.33 | platelet-derived growth factor alpha polypeptide |
| 202113_s_at | SNX2 | 404.6 | 942.4 | -2.33 | sorting nexin 2 |
| 215464_s_at | TAX1BP3 | 268.2 | 560.2 | -2.33 | Tax1 (human T-cell leukemia virus type I) binding protein 3 |
| 226084_at | MAP1B | 130.0 | 332.9 | -2.35 | microtubule-associated protein 1B |
| 215236_s_at | PICALM | 101.1 | 236.7 | -2.35 | phosphatidylinositol binding clathrin assembly protein |
| 200695_at | PPP2R1A | 119.5 | 240.5 | -2.35 | protein phosphatase 2 (formerly 2A), regulatory subunit A, alpha isoform |
| 228461_at | SH3RF3 | 115.3 | 343.5 | -2.35 | SH3 domain containing ring finger 3 |
| 221485_at | B4GALT5 | 211.8 | 510.9 | -2.37 | UDP-Gal:betaGlcNAc beta 1,4- galactosyltransferase, polypeptide 5 |
| 223068_at | EML4 | 173.7 | 444.1 | -2.37 | echinoderm microtubule associated protein like 4 |
| 200634_at | PFN1 | 555.3 | 1318.3 | -2.37 | profilin 1 |
| 204359_at | FLRT2 | 503.1 | 1195.6 | -2.39 | fibronectin leucine rich transmembrane protein 2 |
| 203233_at | IL4R | 166.0 | 460.3 | -2.39 | interleukin 4 receptor |
| 226001_at | KLHL5 | 217.7 | 563.9 | -2.39 | kelch-like 5 (Drosophila) |
| 218036_x_at | NMD3 | 120.8 | 298.3 | -2.39 | NMD3 homolog (S. cerevisiae) |
| 212680_x_at | PPP1R14B | 270.5 | 512.1 | -2.39 | protein phosphatase 1, regulatory (inhibitor) subunit 14B |
| 201617_x_at | CALD1 | 573.3 | 1172.8 | -2.41 | caldesmon 1 |
| 203234_at | UPP1 | 61.1 | 178.7 | -2.41 | uridine phosphorylase 1 |
| 225436_at | FAM108C1 | 53.2 | 138.0 | -2.42 | family with sequence similarity 108, member C1 |
| 203397_s_at | GALNT3 | 59.8 | 187.3 | -2.42 | UDP-N-acetyl-alpha-D-galactosamine:polypeptide N-acetylgalactosaminyltransferase 3 (GalNAc-T3) |
| 218486_at | KLF11 | 222.9 | 554.4 | -2.42 | Kruppel-like factor 11 |
| 203320_at | SH2B3 | 199.8 | 535.3 | -2.42 | SH2B adaptor protein 3 |
| 219770_at | GTDC1 | 101.2 | 255.7 | -2.44 | glycosyltransferase-like domain containing 1 |
| 204897_at | PTGER4 | 209.6 | 591.1 | -2.44 | prostaglandin E receptor 4 (subtype EP4) |
| 209543_s_at | CD34 | 114.3 | 271.3 | -2.46 | CD34 molecule |
| 201809_s_at | ENG | 463.9 | 1100.3 | -2.46 | endoglin |
| 231577_s_at | GBP1 | 76.8 | 202.8 | -2.46 | guanylate binding protein 1, interferon-inducible, 67kDa |
| 227314_at | ITGA2 | 133.3 | 450.1 | -2.46 | integrin, alpha 2 (CD49B, alpha 2 subunit of VLA-2 receptor) |
| 218039_at | NUSAP1 | 82.5 | 214.8 | -2.46 | nucleolar and spindle associated protein 1 |
| 225585_at | RAP2A | 351.3 | 813.5 | -2.46 | RAP2A, member of RAS oncogene family |
| 223533_at | LRRC8C | 143.3 | 270.4 | -2.48 | leucine rich repeat containing 8 family, member C |
| 201058_s_at | MYL9 | 197.8 | 472.8 | -2.48 | myosin, light chain 9, regulatory |
| 222752_s_at | TMEM206 | 114.0 | 311.1 | -2.48 | transmembrane protein 206 |
| 209946_at | VEGFC | 102.1 | 214.8 | -2.48 | vascular endothelial growth factor C |
| 210186_s_at | FKBP1A | 192.0 | 490.3 | -2.50 | FK506 binding protein 1A, 12kDa |
| 214453_s_at | IFI44 | 169.1 | 357.8 | -2.50 | interferon-induced protein 44 |
| 1552264_a_at | MAPK1 | 54.7 | 132.6 | -2.50 | mitogen-activated protein kinase 1 |
| 202273_at | PDGFRB | 406.2 | 1212.9 | -2.50 | platelet-derived growth factor receptor, beta polypeptide |
| 208690_s_at | PDLIM1 | 722.7 | 1838.5 | -2.50 | PDZ and LIM domain 1 |
| 202820_at | AHR | 301.7 | 635.0 | -2.52 | aryl hydrocarbon receptor |
| 204345_at | COL16A1 | 524.7 | 1453.8 | -2.52 | collagen, type XVI, alpha 1 |
| 211958_at | IGFBP5 | 174.2 | 358.0 | -2.52 | insulin-like growth factor binding protein 5 |
| 47560_at | LPHN1 | 77.8 | 175.2 | -2.54 | latrophilin 1 |
| 204079_at | TPST2 | 190.8 | 594.7 | -2.54 | tyrosylprotein sulfotransferase 2 |
| 209228_x_at | TUSC3 | 136.0 | 299.6 | -2.54 | tumor suppressor candidate 3 |
| 209765_at | ADAM19 | 46.0 | 149.5 | -2.56 | ADAM metallopeptidase domain 19 (meltrin beta) |
| 1558501_at | DNM3 | 79.3 | 193.4 | -2.56 | dynamin 3 |
| 201995_at | EXT1 | 203.0 | 732.0 | -2.56 | exostoses (multiple) 1 |
| 202765_s_at | FBN1 | 226.3 | 537.9 | -2.56 | fibrillin 1 |
| 212070_at | GPR56 | 183.5 | 364.5 | -2.56 | G protein-coupled receptor 56 |
| 232080_at | HECW2 | 234.1 | 531.1 | -2.56 | HECT, C2 and WW domain containing E3 ubiquitin protein ligase 2 |
| 209200_at | MEF2C | 396.7 | 1057.9 | -2.56 | myocyte enhancer factor 2C |
| 225790_at | MSRB3 | 152.8 | 348.4 | -2.56 | methionine sulfoxide reductase B3 |
| 218009_s_at | PRC1 | 76.7 | 181.9 | -2.56 | protein regulator of cytokinesis 1 |
| 230730_at | SGCD | 134.8 | 307.5 | -2.56 | sarcoglycan, delta (35kDa dystrophin-associated glycoprotein) |
| 221276_s_at | SYNC | 72.4 | 198.2 | -2.56 | syncoilin, intermediate filament protein |
| 205990_s_at | WNT5A | 38.7 | 104.5 | -2.56 | wingless-type MMTV integration site family, member 5A |
| 228402_at | ZBED3 | 67.2 | 142.5 | -2.56 | zinc finger, BED-type containing 3 |
| 204567_s_at | ABCG1 | 90.4 | 201.3 | -2.58 | ATP-binding cassette, sub-family G (WHITE), member 1 |
| 226499_at | NRARP | 135.1 | 440.7 | -2.58 | NOTCH-regulated ankyrin repeat protein |
| 219148_at | PBK | 28.7 | 64.2 | -2.58 | PDZ binding kinase |
| 214435_x_at | RALA | 170.0 | 428.6 | -2.58 | v-ral simian leukemia viral oncogene homolog A (ras related) |
| 213293_s_at | TRIM22 | 772.2 | 1397.3 | -2.58 | tripartite motif-containing 22 |
| 205257_s_at | AMPH | 70.4 | 193.9 | -2.60 | amphiphysin |
| 203358_s_at | EZH2 | 42.1 | 104.9 | -2.60 | enhancer of zeste homolog 2 (Drosophila) |
| 217755_at | HN1 | 85.8 | 230.9 | -2.60 | hematological and neurological expressed 1 |
| 200798_x_at | MCL1 | 827.6 | 1934.3 | -2.60 | myeloid cell leukemia sequence 1 (BCL2-related) |
| 208078_s_at | SIK1 | 316.2 | 780.9 | -2.60 | salt-inducible kinase 1 |
| 225524_at | ANTXR2 | 398.1 | 1016.2 | -2.62 | anthrax toxin receptor 2 |
| 209121_x_at | NR2F2 | 389.3 | 1016.7 | -2.62 | nuclear receptor subfamily 2, group F, member 2 |
| 1552485_at | LACTB | 39.6 | 116.8 | -2.64 | lactamase, beta |
| 204040_at | RNF144A | 211.1 | 525.3 | -2.64 | ring finger protein 144A |
| 226899_at | UNC5B | 184.2 | 545.2 | -2.64 | unc-5 homolog B (C. elegans) |
| 212067_s_at | C1R | 596.9 | 1796.4 | -2.66 | complement component 1, r subcomponent |
| 202435_s_at | CYP1B1 | 786.7 | 1831.7 | -2.66 | cytochrome P450, family 1, subfamily B, polypeptide 1 |
| 223875_s_at | EPC1 | 71.0 | 137.7 | -2.66 | enhancer of polycomb homolog 1 (Drosophila) |
| 226525_at | STK17B | 291.6 | 570.5 | -2.66 | serine/threonine kinase 17b |
| 232914_s_at | SYTL2 | 357.2 | 742.5 | -2.66 | synaptotagmin-like 2 |
| 218113_at | TMEM2 | 311.3 | 832.6 | -2.66 | transmembrane protein 2 |
| 209081_s_at | COL18A1 | 357.2 | 860.9 | -2.68 | collagen, type XVIII, alpha 1 |
| 203499_at | EPHA2 | 123.9 | 357.3 | -2.68 | EPH receptor A2 |
| 217763_s_at | RAB31 | 552.9 | 1318.1 | -2.68 | RAB31, member RAS oncogene family |
| 210042_s_at | CTSZ | 215.4 | 584.6 | -2.70 | cathepsin Z |
| 203888_at | THBD | 252.7 | 590.2 | -2.70 | thrombomodulin |
| 224823_at | MYLK | 738.6 | 1673.5 | -2.72 | myosin light chain kinase |
| 221031_s_at | APOLD1 | 616.3 | 1187.2 | -2.74 | apolipoprotein L domain containing 1 |
| 202450_s_at | CTSK | 2527.9 | 7594.7 | -2.74 | cathepsin K |
| 238756_at | GAS2L3 | 71.5 | 183.2 | -2.74 | Growth arrest-specific 2 like 3 |
| 216041_x_at | GRN | 528.7 | 1662.9 | -2.74 | granulin |
| 209949_at | NCF2 | 29.6 | 62.5 | -2.74 | neutrophil cytosolic factor 2 |
| 206303_s_at | NUDT4 | 91.3 | 187.4 | -2.74 | nudix (nucleoside diphosphate linked moiety X)-type motif 4 |
| 225303_at | KIRREL | 155.1 | 565.2 | -2.76 | kin of IRRE like (Drosophila) |
| 211762_s_at | KPNA2 | 195.0 | 564.3 | -2.76 | karyopherin alpha 2 (RAG cohort 1, importin alpha 1) |
| 201387_s_at | UCHL1 | 73.6 | 199.7 | -2.76 | ubiquitin carboxyl-terminal esterase L1 (ubiquitin thiolesterase) |
| 201089_at | ATP6V1B2 | 419.0 | 1110.7 | -2.79 | ATPase, H+ transporting, lysosomal 56/58kDa, V1 subunit B2 |
| 226853_at | BMP2K | 121.0 | 291.1 | -2.79 | BMP2 inducible kinase |
| 224994_at | CAMK2D | 122.7 | 351.5 | -2.79 | calcium/calmodulin-dependent protein kinase II delta |
| 212771_at | FAM171A1 | 299.8 | 679.0 | -2.79 | family with sequence similarity 171, member A1 |
| 204527_at | MYO5A | 91.7 | 245.7 | -2.79 | myosin VA (heavy chain 12, myoxin) |
| 209040_s_at | PSMB8 | 184.8 | 415.9 | -2.79 | proteasome (prosome, macropain) subunit, beta type, 8 (large multifunctional peptidase 7) |
| 208637_x_at | ACTN1 | 351.2 | 1003.8 | -2.81 | actinin, alpha 1 |
| 211395_x_at | FCGR2C | 62.6 | 196.8 | -2.81 | Fc fragment of IgG, low affinity IIc, receptor for (CD32) |
| 211656_x_at | HLA-DQB1 /// LOC100294318 | 175.1 | 446.4 | -2.81 | major histocompatibility complex, class II, DQ beta 1 /// similar to major histocompatibility complex, class II, DQ beta 1 |
| 203570_at | LOXL1 | 150.3 | 502.1 | -2.81 | lysyl oxidase-like 1 |
| 207542_s_at | AQP1 /// INMT | 534.9 | 1485.4 | -2.83 | aquaporin 1 (Colton blood group) /// indolethylamine N-methyltransferase |
| 204821_at | BTN3A3 | 86.8 | 244.5 | -2.83 | butyrophilin, subfamily 3, member A3 |
| 1554966_a_at | FILIP1L | 189.5 | 443.1 | -2.83 | filamin A interacting protein 1-like |
| 205609_at | ANGPT1 | 121.3 | 274.3 | -2.85 | angiopoietin 1 |
| 219787_s_at | ECT2 | 46.7 | 145.7 | -2.85 | epithelial cell transforming sequence 2 oncogene |
| 219656_at | PCDH12 | 113.0 | 328.9 | -2.85 | protocadherin 12 |
| 210993_s_at | SMAD1 | 217.6 | 629.2 | -2.85 | SMAD family member 1 |
| 41037_at | TEAD4 | 38.2 | 91.7 | -2.85 | TEA domain family member 4 |
| 210643_at | TNFSF11 | 353.4 | 1047.9 | -2.85 | tumor necrosis factor (ligand) superfamily, member 11 |
| 227923_at | SHANK3 | 172.7 | 460.5 | -2.87 | SH3 and multiple ankyrin repeat domains 3 |
| 213548_s_at | CDV3 | 29.7 | 70.7 | -2.89 | CDV3 homolog (mouse) |
| 206116_s_at | TPM1 | 508.6 | 1412.0 | -2.89 | tropomyosin 1 (alpha) |
| 1554547_at | FAM13C | 159.3 | 442.9 | -2.92 | family with sequence similarity 13, member C |
| 217478_s_at | HLA-DMA /// HLA-DMB | 254.4 | 701.4 | -2.92 | major histocompatibility complex, class II, DM alpha /// major histocompatibility complex, class II, DM beta |
| 203417_at | MFAP2 | 193.5 | 661.5 | -2.92 | microfibrillar-associated protein 2 |
| 228964_at | PRDM1 | 79.0 | 267.5 | -2.92 | PR domain containing 1, with ZNF domain |
| 202497_x_at | SLC2A3 | 147.4 | 401.1 | -2.92 | solute carrier family 2 (facilitated glucose transporter), member 3 |
| 202465_at | PCOLCE | 1115.2 | 3949.8 | -2.94 | procollagen C-endopeptidase enhancer |
| 202589_at | TYMS | 180.4 | 554.9 | -2.94 | thymidylate synthetase |
| 223499_at | C1QTNF5 /// MFRP | 358.4 | 1126.6 | -2.96 | C1q and tumor necrosis factor related protein 5 /// membrane frizzled-related protein |
| 33767_at | NEFH | 21.5 | 55.2 | -2.96 | neurofilament, heavy polypeptide |
| 224848_at | CDK6 | 96.0 | 235.1 | -2.99 | cyclin-dependent kinase 6 |
| 202718_at | IGFBP2 | 41.9 | 157.6 | -2.99 | insulin-like growth factor binding protein 2, 36kDa |
| 218883_s_at | MLF1IP | 56.7 | 133.6 | -2.99 | MLF1 interacting protein |
| 226490_at | NHSL1 | 116.0 | 343.0 | -2.99 | NHS-like 1 |
| 213222_at | PLCB1 | 233.1 | 634.5 | -2.99 | phospholipase C, beta 1 (phosphoinositide-specific) |
| 203680_at | PRKAR2B | 89.8 | 301.9 | -2.99 | protein kinase, cAMP-dependent, regulatory, type II, beta |
| 208850_s_at | THY1 | 205.3 | 776.5 | -2.99 | Thy-1 cell surface antigen |
| 205392_s_at | CCL14 /// CCL14-CCL15 /// CCL15 | 690.3 | 1626.1 | -3.01 | chemokine (C-C motif) ligand 14 /// chemokine ligand 14, chemokine ligand 15 transcription unit /// chemokine (C-C motif) ligand 15 |
| 209955_s_at | FAP | 544.7 | 1884.0 | -3.01 | fibroblast activation protein, alpha |
| 232024_at | GIMAP2 | 142.5 | 370.4 | -3.01 | GTPase, IMAP family member 2 |
| 215933_s_at | HHEX | 85.6 | 196.4 | -3.01 | hematopoietically expressed homeobox |
| 205206_at | KAL1 | 135.2 | 377.7 | -3.01 | Kallmann syndrome 1 sequence |
| 225613_at | MAST4 | 207.8 | 492.0 | -3.01 | microtubule associated serine/threonine kinase family member 4 |
| 220952_s_at | PLEKHA5 | 258.3 | 1019.9 | -3.01 | pleckstrin homology domain containing, family A member 5 |
| 222803_at | PRTFDC1 | 110.4 | 333.4 | -3.01 | phosphoribosyl transferase domain containing 1 |
| 212646_at | RFTN1 | 476.4 | 1624.0 | -3.01 | raftlin, lipid raft linker 1 |
| 201416_at | SOX4 | 288.9 | 1043.5 | -3.01 | SRY (sex determining region Y)-box 4 |
| 214164_x_at | CA12 | 73.9 | 195.1 | -3.03 | carbonic anhydrase XII |
| 203903_s_at | HEPH | 144.9 | 371.5 | -3.03 | hephaestin |
| 1553530_a_at | ITGB1 | 531.5 | 1562.1 | -3.03 | integrin, beta 1 (fibronectin receptor, beta polypeptide, antigen CD29 includes MDF2, MSK12) |
| 203835_at | LRRC32 | 149.5 | 511.2 | -3.03 | leucine rich repeat containing 32 |
| 213169_at | SEMA5A | 183.7 | 520.2 | -3.03 | sema domain, seven thrombospondin repeats (type 1 and type 1-like), transmembrane domain (TM) and short cytoplasmic domain, (semaphorin) 5A |
| 228698_at | SOX7 | 277.1 | 708.8 | -3.03 | SRY (sex determining region Y)-box 7 |
| 214587_at | COL8A1 | 59.0 | 166.6 | -3.05 | collagen, type VIII, alpha 1 |
| 202409_at | IGF2 /// INS-IGF2 | 483.0 | 1539.7 | -3.05 | insulin-like growth factor 2 (somatomedin A) /// INS-IGF2 readthrough transcript |
| 206765_at | KCNJ2 | 56.2 | 262.0 | -3.05 | potassium inwardly-rectifying channel, subfamily J, member 2 |
| 204446_s_at | ALOX5 | 64.6 | 205.6 | -3.08 | arachidonate 5-lipoxygenase |
| 234994_at | TMEM200A | 75.7 | 261.5 | -3.08 | transmembrane protein 200A |
| 210609_s_at | TP53I3 | 111.2 | 309.4 | -3.08 | tumor protein p53 inducible protein 3 |
| 228570_at | BTBD11 | 47.8 | 156.0 | -3.10 | BTB (POZ) domain containing 11 |
| 229218_at | COL1A2 | 983.2 | 3317.2 | -3.10 | collagen, type I, alpha 2 |
| 212489_at | COL5A1 | 918.3 | 2930.4 | -3.10 | collagen, type V, alpha 1 |
| 227609_at | EPSTI1 | 44.5 | 147.9 | -3.10 | epithelial stromal interaction 1 (breast) |
| 210889_s_at | FCGR2B | 47.3 | 171.4 | -3.10 | Fc fragment of IgG, low affinity IIb, receptor (CD32) |
| 204220_at | GMFG | 140.0 | 497.7 | -3.10 | glia maturation factor, gamma |
| 203414_at | MMD | 120.9 | 377.2 | -3.10 | monocyte to macrophage differentiation-associated |
| 222162_s_at | ADAMTS1 | 528.0 | 1133.5 | -3.13 | ADAM metallopeptidase with thrombospondin type 1 motif, 1 |
| 202888_s_at | ANPEP | 181.3 | 646.4 | -3.13 | alanyl (membrane) aminopeptidase |
| 211126_s_at | CSRP2 | 91.2 | 239.8 | -3.13 | cysteine and glycine-rich protein 2 |
| 211368_s_at | CASP1 | 56.7 | 208.0 | -3.15 | caspase 1, apoptosis-related cysteine peptidase (interleukin 1, beta, convertase) |
| 221541_at | CRISPLD2 | 646.9 | 2234.8 | -3.15 | cysteine-rich secretory protein LCCL domain containing 2 |
| 201858_s_at | SRGN | 359.5 | 924.7 | -3.15 | serglycin |
| 202643_s_at | TNFAIP3 | 82.5 | 292.0 | -3.15 | tumor necrosis factor, alpha-induced protein 3 |
| 202705_at | CCNB2 | 17.7 | 90.1 | -3.17 | cyclin B2 |
| 229800_at | DCLK1 | 68.9 | 225.3 | -3.17 | Doublecortin-like kinase 1 |
| 203434_s_at | MME | 32.5 | 109.9 | -3.17 | membrane metallo-endopeptidase |
| 225688_s_at | PHLDB2 | 397.3 | 1000.8 | -3.17 | pleckstrin homology-like domain, family B, member 2 |
| 202756_s_at | GPC1 | 112.3 | 310.9 | -3.20 | glypican 1 |
| 213258_at | TFPI | 337.0 | 886.9 | -3.20 | tissue factor pathway inhibitor (lipoprotein-associated coagulation inhibitor) |
| 218975_at | COL5A3 | 63.9 | 197.4 | -3.22 | collagen, type V, alpha 3 |
| 204780_s_at | FAS | 110.0 | 294.0 | -3.22 | Fas (TNF receptor superfamily, member 6) |
| 223434_at | GBP3 | 180.0 | 638.4 | -3.22 | guanylate binding protein 3 |
| 228776_at | GJC1 | 450.7 | 1053.4 | -3.22 | gap junction protein, gamma 1, 45kDa |
| 219793_at | SNX16 | 39.8 | 90.8 | -3.22 | sorting nexin 16 |
| 225987_at | STEAP4 | 447.2 | 1962.3 | -3.22 | STEAP family member 4 |
| 219059_s_at | LYVE1 | 60.7 | 224.4 | -3.25 | lymphatic vessel endothelial hyaluronan receptor 1 |
| 219700_at | PLXDC1 | 181.5 | 666.8 | -3.25 | plexin domain containing 1 |
| 201971_s_at | ATP6V1A | 31.2 | 124.3 | -3.27 | ATPase, H+ transporting, lysosomal 70kDa, V1 subunit A |
| 212486_s_at | FYN | 82.4 | 244.7 | -3.27 | FYN oncogene related to SRC, FGR, YES |
| 213975_s_at | LYZ | 406.2 | 1284.4 | -3.27 | lysozyme (renal amyloidosis) |
| 227210_at | SFMBT2 | 63.0 | 182.7 | -3.27 | Scm-like with four mbt domains 2 |
| 209619_at | CD74 | 407.8 | 1786.4 | -3.30 | CD74 molecule, major histocompatibility complex, class II invariant chain |
| 212192_at | KCTD12 | 1441.9 | 4359.9 | -3.30 | potassium channel tetramerisation domain containing 12 |
| 201847_at | LIPA | 660.4 | 1771.8 | -3.30 | lipase A, lysosomal acid, cholesterol esterase |
| 217177_s_at | PTPRB | 73.0 | 136.6 | -3.30 | protein tyrosine phosphatase, receptor type, B |
| 1558254_s_at | SRPK2 | 85.3 | 232.3 | -3.30 | SFRS protein kinase 2 |
| 207556_s_at | DGKZ | 54.1 | 172.7 | -3.32 | diacylglycerol kinase, zeta 104kDa |
| 209152_s_at | TCF3 | 36.6 | 82.9 | -3.32 | transcription factor 3 (E2A immunoglobulin enhancer binding factors E12/E47) |
| 210657_s_at | SEPT4 | 108.2 | 293.4 | -3.35 | septin 4 |
| 219134_at | ELTD1 | 348.4 | 960.5 | -3.38 | EGF, latrophilin and seven transmembrane domain containing 1 |
| 209098_s_at | JAG1 | 170.5 | 465.3 | -3.38 | jagged 1 (Alagille syndrome) |
| 211864_s_at | MYOF | 176.5 | 779.6 | -3.38 | myoferlin |
| 209457_at | DUSP5 | 112.2 | 442.9 | -3.40 | dual specificity phosphatase 5 |
| 235371_at | GLT8D4 | 184.9 | 707.4 | -3.40 | glycosyltransferase 8 domain containing 4 |
| 213170_at | GPX7 | 140.4 | 492.0 | -3.43 | glutathione peroxidase 7 |
| 212951_at | GPR116 | 156.7 | 441.2 | -3.46 | G protein-coupled receptor 116 |
| 201508_at | IGFBP4 | 141.8 | 539.4 | -3.46 | insulin-like growth factor binding protein 4 |
| 212235_at | PLXND1 | 124.8 | 441.8 | -3.46 | plexin D1 |
| 224937_at | PTGFRN | 230.4 | 745.1 | -3.46 | prostaglandin F2 receptor negative regulator |
| 205269_at | LCP2 | 49.3 | 182.0 | -3.48 | lymphocyte cytosolic protein 2 (SH2 domain containing leukocyte protein of 76kDa) |
| 227911_at | ARHGAP28 | 56.4 | 168.5 | -3.51 | Rho GTPase activating protein 28 |
| 202760_s_at | PALM2-AKAP2 | 296.2 | 749.5 | -3.51 | PALM2-AKAP2 readthrough transcript |
| 231823_s_at | SH3PXD2B | 168.2 | 805.1 | -3.51 | SH3 and PX domains 2B |
| 233085_s_at | OBFC2A | 34.2 | 153.9 | -3.54 | oligonucleotide/oligosaccharide-binding fold containing 2A |
| 202759_s_at | AKAP2 /// PALM2-AKAP2 | 493.6 | 1319.4 | -3.56 | A kinase (PRKA) anchor protein 2 /// PALM2-AKAP2 readthrough transcript |
| 225166_at | ARHGAP18 | 33.3 | 102.6 | -3.56 | Rho GTPase activating protein 18 |
| 203921_at | CHST2 | 77.8 | 193.8 | -3.56 | carbohydrate (N-acetylglucosamine-6-O) sulfotransferase 2 |
| 209286_at | CDC42EP3 | 124.5 | 391.4 | -3.59 | CDC42 effector protein (Rho GTPase binding) 3 |
| 203474_at | IQGAP2 | 49.2 | 156.1 | -3.59 | IQ motif containing GTPase activating protein 2 |
| 212588_at | PTPRC | 67.6 | 335.9 | -3.59 | protein tyrosine phosphatase, receptor type, C |
| 235593_at | ZEB2 | 55.6 | 224.7 | -3.59 | zinc finger E-box binding homeobox 2 |
| 201141_at | GPNMB | 1309.8 | 3827.1 | -3.62 | glycoprotein (transmembrane) nmb |
| 204232_at | FCER1G | 157.1 | 486.5 | -3.65 | Fc fragment of IgE, high affinity I, receptor for; gamma polypeptide |
| 229055_at | GPR68 | 64.7 | 256.7 | -3.65 | G protein-coupled receptor 68 |
| 226992_at | NOSTRIN | 105.7 | 264.0 | -3.65 | nitric oxide synthase trafficker |
| 203504_s_at | ABCA1 | 87.1 | 360.1 | -3.68 | ATP-binding cassette, sub-family A (ABC1), member 1 |
| 226824_at | CPXM2 | 193.4 | 802.8 | -3.68 | carboxypeptidase X (M14 family), member 2 |
| 202202_s_at | LAMA4 | 572.9 | 1667.4 | -3.68 | laminin, alpha 4 |
| 206571_s_at | MAP4K4 | 162.6 | 655.7 | -3.68 | mitogen-activated protein kinase kinase kinase kinase 4 |
| 205326_at | RAMP3 | 148.1 | 557.1 | -3.68 | receptor (G protein-coupled) activity modifying protein 3 |
| 205195_at | AP1S1 | 27.4 | 86.4 | -3.70 | adaptor-related protein complex 1, sigma 1 subunit |
| 225258_at | FBLIM1 | 108.9 | 354.3 | -3.70 | filamin binding LIM protein 1 |
| 225167_at | FRMD4A | 49.1 | 125.7 | -3.70 | FERM domain containing 4A |
| 204912_at | IL10RA | 69.6 | 312.0 | -3.70 | interleukin 10 receptor, alpha |
| 213241_at | PLXNC1 | 61.6 | 231.7 | -3.70 | plexin C1 |
| 227399_at | VGLL3 | 186.3 | 434.7 | -3.70 | vestigial like 3 (Drosophila) |
| 212865_s_at | COL14A1 | 809.7 | 2209.8 | -3.73 | collagen, type XIV, alpha 1 |
| 202295_s_at | CTSH | 241.7 | 1091.1 | -3.73 | cathepsin H |
| 203923_s_at | CYBB | 83.6 | 364.6 | -3.73 | cytochrome b-245, beta polypeptide |
| 203471_s_at | PLEK | 48.3 | 140.7 | -3.73 | pleckstrin |
| 202668_at | EFNB2 | 368.4 | 675.4 | -3.76 | ephrin-B2 |
| 222088_s_at | SLC2A14 /// SLC2A3 | 135.5 | 422.9 | -3.76 | solute carrier family 2 (facilitated glucose transporter), member 14 /// solute carrier family 2 (facilitated glucose transporter), member 3 |
| 225834_at | FAM72A /// FAM72B /// FAM72C /// FAM72D | 40.3 | 175.8 | -3.79 | family with sequence similarity 72, member A /// family with sequence similarity 72, member B /// family with sequence similarity 72, member C /// family with sequence similarity 72, member D |
| 201137_s_at | HLA-DPB1 | 760.4 | 2869.7 | -3.79 | major histocompatibility complex, class II, DP beta 1 |
| 212143_s_at | IGFBP3 | 247.5 | 778.2 | -3.79 | insulin-like growth factor binding protein 3 |
| 213479_at | NPTX2 | 130.8 | 380.4 | -3.79 | neuronal pentraxin II |
| 203185_at | RASSF2 | 70.6 | 281.4 | -3.79 | Ras association (RalGDS/AF-6) domain family member 2 |
| 226436_at | RASSF4 | 102.1 | 447.2 | -3.79 | Ras association (RalGDS/AF-6) domain family member 4 |
| 214844_s_at | DOK5 | 98.7 | 439.0 | -3.85 | docking protein 5 |
| 225355_at | NEURL1B | 352.6 | 985.6 | -3.85 | neuralized homolog 1B (Drosophila) |
| 206702_at | TEK | 212.9 | 553.2 | -3.85 | TEK tyrosine kinase, endothelial |
| 227870_at | IGDCC4 | 189.4 | 657.8 | -3.88 | immunoglobulin superfamily, DCC subclass, member 4 |
| 209541_at | IGF1 | 178.1 | 898.5 | -3.88 | insulin-like growth factor 1 (somatomedin C) |
| 212956_at | TBC1D9 | 199.5 | 841.5 | -3.88 | TBC1 domain family, member 9 (with GRAM domain) |
| 211709_s_at | CLEC11A | 402.4 | 1627.2 | -3.91 | C-type lectin domain family 11, member A |
| 226817_at | DSC2 | 55.7 | 255.2 | -3.91 | desmocollin 2 |
| 201667_at | GJA1 | 1928.9 | 6780.6 | -3.91 | gap junction protein, alpha 1, 43kDa |
| 214500_at | H2AFY | 70.3 | 214.8 | -3.91 | H2A histone family, member Y |
| 218162_at | OLFML3 | 378.2 | 1925.3 | -3.91 | olfactomedin-like 3 |
| 1555789_s_at | PHF23 | 33.9 | 162.3 | -3.91 | PHD finger protein 23 |
| 231947_at | MYCT1 | 298.0 | 618.4 | -3.97 | myc target 1 |
| 202283_at | SERPINF1 | 1822.9 | 7398.3 | -3.97 | serpin peptidase inhibitor, clade F (alpha-2 antiplasmin, pigment epithelium derived factor), member 1 |
| 211965_at | ZFP36L1 | 72.2 | 198.2 | -3.97 | zinc finger protein 36, C3H type-like 1 |
| 227812_at | TNFRSF19 | 110.8 | 462.8 | -4.00 | tumor necrosis factor receptor superfamily, member 19 |
| 202957_at | HCLS1 | 109.0 | 480.6 | -4.03 | hematopoietic cell-specific Lyn substrate 1 |
| 210839_s_at | ENPP2 | 184.4 | 920.0 | -4.09 | ectonucleotide pyrophosphatase/phosphodiesterase 2 |
| 213247_at | SVEP1 | 107.7 | 511.5 | -4.09 | sushi, von Willebrand factor type A, EGF and pentraxin domain containing 1 |
| 208096_s_at | COL21A1 | 156.6 | 756.9 | -4.13 | collagen, type XXI, alpha 1 |
| 206481_s_at | LDB2 | 372.7 | 1218.7 | -4.13 | LIM domain binding 2 |
| 214770_at | MSR1 | 54.0 | 208.0 | -4.13 | macrophage scavenger receptor 1 |
| 244840_x_at | DOCK4 | 36.2 | 107.0 | -4.16 | dedicator of cytokinesis 4 |
| 203868_s_at | VCAM1 | 221.9 | 1177.6 | -4.16 | vascular cell adhesion molecule 1 |
| 222608_s_at | ANLN | 34.5 | 153.6 | -4.19 | anillin, actin binding protein |
| 230422_at | FPR3 | 41.2 | 158.5 | -4.19 | formyl peptide receptor 3 |
| 201785_at | RNASE1 | 495.7 | 1901.7 | -4.19 | ribonuclease, RNase A family, 1 (pancreatic) |
| 53991_at | DENND2A | 24.1 | 86.4 | -4.22 | DENN/MADD domain containing 2A |
| 219032_x_at | OPN3 | 89.6 | 400.2 | -4.22 | opsin 3 |
| 205479_s_at | PLAU | 84.5 | 582.5 | -4.22 | plasminogen activator, urokinase |
| 204286_s_at | PMAIP1 | 21.2 | 130.1 | -4.22 | phorbol-12-myristate-13-acetate-induced protein 1 |
| 224817_at | SH3PXD2A | 872.3 | 1874.1 | -4.22 | SH3 and PX domains 2A |
| 209546_s_at | APOL1 | 60.2 | 150.4 | -4.25 | apolipoprotein L, 1 |
| 219683_at | FZD3 | 15.9 | 66.1 | -4.25 | frizzled homolog 3 (Drosophila) |
| 205419_at | GPR183 | 56.6 | 426.5 | -4.25 | G protein-coupled receptor 183 |
| 218711_s_at | SDPR | 18.6 | 79.2 | -4.25 | serum deprivation response (phosphatidylserine binding protein) |
| 202546_at | VAMP8 | 108.1 | 512.8 | -4.25 | vesicle-associated membrane protein 8 (endobrevin) |
| 224583_at | COTL1 | 172.4 | 813.5 | -4.29 | coactosin-like 1 (Dictyostelium) |
| 228176_at | S1PR3 | 376.3 | 1229.4 | -4.29 | sphingosine-1-phosphate receptor 3 |
| 218585_s_at | DTL | 21.6 | 70.0 | -4.32 | denticleless homolog (Drosophila) |
| 209773_s_at | RRM2 | 31.9 | 159.2 | -4.32 | ribonucleotide reductase M2 |
| 230061_at | TM4SF18 | 189.7 | 546.0 | -4.42 | Transmembrane 4 L six family member 18 |
| 227779_at | ECSCR | 200.0 | 641.3 | -4.46 | Endothelial cell-specific chemotaxis regulator |
| 232136_s_at | CTTNBP2 | 18.5 | 54.4 | -4.52 | cortactin binding protein 2 |
| 218223_s_at | PLEKHO1 | 35.5 | 236.9 | -4.52 | pleckstrin homology domain containing, family O member 1 |
| 204070_at | RARRES3 | 117.7 | 532.0 | -4.52 | retinoic acid receptor responder (tazarotene induced) 3 |
| 209030_s_at | CADM1 | 146.8 | 670.8 | -4.56 | cell adhesion molecule 1 |
| 206488_s_at | CD36 | 172.9 | 892.8 | -4.59 | CD36 molecule (thrombospondin receptor) |
| 206710_s_at | EPB41L3 | 71.5 | 205.8 | -4.59 | erythrocyte membrane protein band 4.1-like 3 |
| 225464_at | FRMD6 | 418.5 | 1987.4 | -4.59 | FERM domain containing 6 |
| 202992_at | C7 | 134.2 | 502.3 | -4.67 | complement component 7 |
| 227242_s_at | EBF3 | 76.9 | 327.6 | -4.67 | early B-cell factor 3 |
| 204984_at | GPC4 | 79.5 | 433.9 | -4.67 | glypican 4 |
| 213419_at | APBB2 | 57.1 | 179.1 | -4.70 | amyloid beta (A4) precursor protein-binding, family B, member 2 |
| 207828_s_at | CENPF | 25.7 | 102.3 | -4.70 | centromere protein F, 350/400ka (mitosin) |
| 1554614_a_at | PTBP2 | 40.2 | 104.5 | -4.70 | polypyrimidine tract binding protein 2 |
| 206868_at | STARD8 | 44.3 | 134.2 | -4.70 | StAR-related lipid transfer (START) domain containing 8 |
| 203817_at | GUCY1B3 | 332.2 | 867.0 | -4.74 | guanylate cyclase 1, soluble, beta 3 |
| 218589_at | LPAR6 | 208.9 | 906.1 | -4.74 | lysophosphatidic acid receptor 6 |
| 226673_at | SH2D3C | 119.1 | 326.3 | -4.74 | SH2 domain containing 3C |
| 225647_s_at | CTSC | 101.9 | 471.2 | -4.78 | cathepsin C |
| 205462_s_at | HPCAL1 | 41.5 | 159.8 | -4.78 | hippocalcin-like 1 |
| 1555564_a_at | CFI | 67.7 | 270.3 | -4.81 | complement factor I |
| 203813_s_at | SLIT3 | 49.2 | 254.7 | -4.85 | slit homolog 3 (Drosophila) |
| 209583_s_at | CD200 | 244.0 | 694.0 | -4.89 | CD200 molecule |
| 208893_s_at | DUSP6 | 112.2 | 503.3 | -4.92 | dual specificity phosphatase 6 |
| 202859_x_at | IL8 | 69.0 | 335.6 | -4.96 | interleukin 8 |
| 1552703_s_at | CARD16 /// CASP1 | 81.5 | 236.8 | -5.00 | caspase recruitment domain family, member 16 /// caspase 1, apoptosis-related cysteine peptidase (interleukin 1, beta, convertase) |
| 225369_at | ESAM | 94.0 | 242.8 | -5.00 | endothelial cell adhesion molecule |
| 204438_at | MRC1 /// MRC1L1 | 76.3 | 425.8 | -5.00 | mannose receptor, C type 1 /// mannose receptor, C type 1-like 1 |
| 38487_at | STAB1 | 156.0 | 630.3 | -5.00 | stabilin 1 |
| 212624_s_at | CHN1 | 149.3 | 862.4 | -5.04 | chimerin (chimaerin) 1 |
| 220301_at | CCDC102B | 44.5 | 247.3 | -5.08 | coiled-coil domain containing 102B |
| 212298_at | NRP1 | 403.3 | 1508.1 | -5.08 | neuropilin 1 |
| 227394_at | NCAM1 | 68.3 | 1034.3 | -5.12 | neural cell adhesion molecule 1 |
| 213909_at | LRRC15 | 532.5 | 2677.4 | -5.16 | leucine rich repeat containing 15 |
| 215074_at | MYO1B | 28.6 | 81.6 | -5.32 | myosin IB |
| 205381_at | LRRC17 | 168.9 | 639.9 | -5.36 | leucine rich repeat containing 17 |
| 202827_s_at | MMP14 | 62.9 | 430.4 | -5.36 | matrix metallopeptidase 14 (membrane-inserted) |
| 213943_at | TWIST1 | 153.7 | 754.6 | -5.36 | twist homolog 1 (Drosophila) |
| 202902_s_at | CTSS | 109.1 | 444.8 | -5.40 | cathepsin S |
| 205174_s_at | QPCT | 47.2 | 211.1 | -5.40 | glutaminyl-peptide cyclotransferase |
| 215193_x_at | HLA-DRB1 /// HLA-DRB3 /// HLA-DRB4 | 476.5 | 2537.9 | -5.44 | major histocompatibility complex, class II, DR beta 1 /// major histocompatibility complex, class II, DR beta 3 /// major histocompatibility complex, class II, DR beta 4 |
| 213894_at | THSD7A | 43.5 | 130.5 | -5.44 | thrombospondin, type I, domain containing 7A |
| 205554_s_at | DNASE1L3 | 303.3 | 1330.0 | -5.49 | deoxyribonuclease I-like 3 |
| 203666_at | CXCL12 | 461.3 | 2751.7 | -5.53 | chemokine (C-X-C motif) ligand 12 (stromal cell-derived factor 1) |
| 202007_at | NID1 | 307.4 | 1255.6 | -5.53 | nidogen 1 |
| 211343_s_at | COL13A1 | 205.6 | 1454.4 | -5.61 | collagen, type XIII, alpha 1 |
| 228167_at | KLHL6 | 23.7 | 67.4 | -5.61 | kelch-like 6 (Drosophila) |
| 201721_s_at | LAPTM5 | 464.8 | 2749.9 | -5.61 | lysosomal protein transmembrane 5 |
| 202877_s_at | CD93 | 94.5 | 405.1 | -5.70 | CD93 molecule |
| 204677_at | CDH5 | 313.3 | 941.5 | -5.74 | cadherin 5, type 2 (vascular endothelium) |
| 208983_s_at | PECAM1 | 307.1 | 993.6 | -5.74 | platelet/endothelial cell adhesion molecule |
| 227289_at | PCDH17 | 101.6 | 349.7 | -5.79 | protocadherin 17 |
| 201939_at | PLK2 | 424.0 | 1082.3 | -5.79 | polo-like kinase 2 (Drosophila) |
| 213592_at | APLNR | 269.7 | 1277.8 | -5.83 | apelin receptor |
| 200783_s_at | STMN1 | 23.8 | 157.9 | -5.83 | stathmin 1 |
| 225353_s_at | C1QC | 111.5 | 555.7 | -5.88 | complement component 1, q subcomponent, C chain |
| 211991_s_at | HLA-DPA1 | 172.7 | 967.5 | -5.88 | major histocompatibility complex, class II, DP alpha 1 |
| 204463_s_at | EDNRA | 36.0 | 123.0 | -5.92 | endothelin receptor type A |
| 209170_s_at | GPM6B | 201.3 | 573.9 | -5.92 | glycoprotein M6B |
| 204051_s_at | SFRP4 | 496.6 | 2665.7 | -5.92 | secreted frizzled-related protein 4 |
| 218559_s_at | MAFB | 722.4 | 3583.0 | -5.97 | v-maf musculoaponeurotic fibrosarcoma oncogene homolog B (avian) |
| 203548_s_at | LPL | 171.8 | 1174.8 | -6.02 | lipoprotein lipase |
| 225681_at | CTHRC1 | 909.5 | 4230.0 | -6.06 | collagen triple helix repeat containing 1 |
| 221814_at | GPR124 | 115.3 | 369.2 | -6.06 | G protein-coupled receptor 124 |
| 91816_f_at | MEX3D | 70.5 | 305.2 | -6.16 | mex-3 homolog D (C. elegans) |
| 209652_s_at | PGF | 75.1 | 276.3 | -6.16 | placental growth factor |
| 227461_at | STON2 | 54.7 | 408.2 | -6.16 | stonin 2 |
| 205159_at | CSF2RB | 69.9 | 338.4 | -6.20 | colony stimulating factor 2 receptor, beta, low-affinity (granulocyte-macrophage) |
| 225897_at | MARCKS | 299.1 | 1416.1 | -6.20 | myristoylated alanine-rich protein kinase C substrate |
| 202345_s_at | FABP5 | 466.3 | 2826.4 | -6.25 | fatty acid binding protein 5 (psoriasis-associated) |
| 227396_at | PTPRJ | 54.5 | 358.5 | -6.25 | protein tyrosine phosphatase, receptor type, J |
| 206496_at | FMO3 | 13.4 | 90.8 | -6.30 | flavin containing monooxygenase 3 |
| 203636_at | MID1 | 56.3 | 201.4 | -6.30 | midline 1 (Opitz/BBB syndrome) |
| 216005_at | TNC | 115.2 | 823.0 | -6.30 | Tenascin C |
| 202112_at | VWF | 291.1 | 1405.1 | -6.30 | von Willebrand factor |
| 204955_at | SRPX | 194.8 | 1435.4 | -6.35 | sushi-repeat-containing protein, X-linked |
| 210815_s_at | CALCRL | 114.0 | 430.8 | -6.40 | calcitonin receptor-like |
| 220460_at | SLCO1C1 | 23.0 | 87.5 | -6.40 | solute carrier organic anion transporter family, member 1C1 |
| 211651_s_at | LAMB1 | 215.7 | 1086.6 | -6.45 | laminin, beta 1 |
| 224356_x_at | MS4A6A | 123.3 | 563.3 | -6.50 | membrane-spanning 4-domains, subfamily A, member 6A |
| 207714_s_at | SERPINH1 | 247.0 | 1761.7 | -6.50 | serpin peptidase inhibitor, clade H (heat shock protein 47), member 1, (collagen binding protein 1) |
| 204122_at | TYROBP | 139.0 | 1063.5 | -6.50 | TYRO protein tyrosine kinase binding protein |
| 201506_at | TGFBI | 928.9 | 6155.6 | -6.60 | transforming growth factor, beta-induced, 68kDa |
| 220532_s_at | TMEM176B | 46.3 | 307.4 | -6.60 | transmembrane protein 176B |
| 217028_at | CXCR4 | 191.5 | 727.9 | -6.70 | chemokine (C-X-C motif) receptor 4 |
| 202075_s_at | PLTP | 94.8 | 406.8 | -6.70 | phospholipid transfer protein |
| 202988_s_at | RGS1 | 31.7 | 252.8 | -6.75 | regulator of G-protein signaling 1 |
| 202391_at | BASP1 | 279.2 | 1549.2 | -6.81 | brain abundant, membrane attached signal protein 1 |
| 204114_at | NID2 | 240.7 | 1809.2 | -6.81 | nidogen 2 (osteonidogen) |
| 220330_s_at | SAMSN1 | 22.7 | 117.5 | -6.81 | SAM domain, SH3 domain and nuclear localization signals 1 |
| 214038_at | CCL8 | 53.9 | 296.8 | -6.86 | chemokine (C-C motif) ligand 8 |
| 224790_at | ASAP1 | 68.9 | 280.2 | -6.91 | ArfGAP with SH3 domain, ankyrin repeat and PH domain 1 |
| 210559_s_at | CDC2 | 10.4 | 78.5 | -6.91 | cell division cycle 2, G1 to S and G2 to M |
| 1555705_a_at | CMTM3 | 90.0 | 427.4 | -6.91 | CKLF-like MARVEL transmembrane domain containing 3 |
| 211966_at | COL4A2 | 75.3 | 465.2 | -6.91 | collagen, type IV, alpha 2 |
| 208894_at | HLA-DRA | 495.0 | 3132.7 | -6.91 | major histocompatibility complex, class II, DR alpha |
| 213125_at | OLFML2B | 386.3 | 2864.7 | -6.96 | olfactomedin-like 2B |
| 227307_at | TSPAN18 | 97.9 | 344.3 | -7.02 | Tetraspanin 18 |
| 231879_at | COL12A1 | 163.8 | 917.3 | -7.07 | collagen, type XII, alpha 1 |
| 205624_at | CPA3 | 30.8 | 248.2 | -7.07 | carboxypeptidase A3 (mast cell) |
| 212187_x_at | PTGDS | 496.2 | 2615.0 | -7.07 | prostaglandin D2 synthase 21kDa (brain) |
| 200644_at | MARCKSL1 | 151.9 | 941.8 | -7.13 | MARCKS-like 1 |
| 209087_x_at | MCAM | 533.3 | 1351.2 | -7.18 | melanoma cell adhesion molecule |
| 207172_s_at | CDH11 | 118.3 | 810.4 | -7.24 | cadherin 11, type 2, OB-cadherin (osteoblast) |
| 211981_at | COL4A1 | 369.6 | 2134.2 | -7.24 | collagen, type IV, alpha 1 |
| 204468_s_at | TIE1 | 183.5 | 385.3 | -7.24 | tyrosine kinase with immunoglobulin-like and EGF-like domains 1 |
| 204787_at | VSIG4 | 54.7 | 274.1 | -7.24 | V-set and immunoglobulin domain containing 4 |
| 205572_at | ANGPT2 | 34.8 | 144.1 | -7.41 | angiopoietin 2 |
| 223620_at | GPR34 | 52.2 | 242.5 | -7.41 | G protein-coupled receptor 34 |
| 225655_at | UHRF1 | 21.4 | 119.6 | -7.41 | ubiquitin-like with PHD and ring finger domains 1 |
| 208146_s_at | CPVL | 37.6 | 225.7 | -7.52 | carboxypeptidase, vitellogenic-like |
| 209774_x_at | CXCL2 | 70.1 | 300.1 | -7.52 | chemokine (C-X-C motif) ligand 2 |
| 227300_at | TMEM119 | 105.9 | 929.2 | -7.58 | transmembrane protein 119 |
| 235044_at | CYYR1 | 101.1 | 320.4 | -7.76 | cysteine/tyrosine-rich 1 |
| 205304_s_at | KCNJ8 | 49.0 | 337.6 | -7.76 | potassium inwardly-rectifying channel, subfamily J, member 8 |
| 212671_s_at | HLA-DQA1 /// HLA-DQA2 /// LOC100294224 /// LOC100294317 | 94.2 | 712.8 | -7.82 | major histocompatibility complex, class II, DQ alpha 1 /// major histocompatibility complex, class II, DQ alpha 2 /// similar to MHC class II antigen /// similar to MHC HLA-DQ alpha |
| 227347_x_at | HES4 | 45.5 | 185.4 | -7.88 | hairy and enhancer of split 4 (Drosophila) |
| 203665_at | HMOX1 | 99.1 | 817.4 | -7.88 | heme oxygenase (decycling) 1 |
| 209035_at | MDK | 10.7 | 136.0 | -8.25 | midkine (neurite growth-promoting factor 2) |
| 213790_at | ADAM12 | 65.9 | 480.2 | -8.44 | ADAM metallopeptidase domain 12 |
| 226814_at | ADAMTS9 | 78.2 | 705.4 | -8.44 | ADAM metallopeptidase with thrombospondin type 1 motif, 9 |
| 204825_at | MELK | 12.2 | 93.9 | -8.44 | maternal embryonic leucine zipper kinase |
| 238846_at | TNFRSF11A | 23.1 | 349.4 | -8.44 | tumor necrosis factor receptor superfamily, member 11a, NFKB activator |
| 213060_s_at | CHI3L2 | 185.8 | 2390.4 | -8.64 | chitinase 3-like 2 |
| 201069_at | MMP2 | 709.7 | 4568.9 | -8.77 | matrix metallopeptidase 2 (gelatinase A, 72kDa gelatinase, 72kDa type IV collagenase) |
| 210220_at | FZD2 | 12.6 | 116.4 | -8.84 | frizzled homolog 2 (Drosophila) |
| 204844_at | ENPEP | 121.0 | 593.6 | -8.91 | glutamyl aminopeptidase (aminopeptidase A) |
| 209474_s_at | ENTPD1 | 49.7 | 200.9 | -8.98 | ectonucleoside triphosphate diphosphohydrolase 1 |
| 219279_at | DOCK10 | 40.7 | 262.6 | -9.33 | dedicator of cytokinesis 10 |
| 226955_at | AFAP1L1 | 49.2 | 246.7 | -9.55 | actin filament associated protein 1-like 1 |
| 206956_at | BGLAP | 79.0 | 3573.7 | -9.77 | bone gamma-carboxyglutamate (gla) protein |
| 1555778_a_at | POSTN | 429.2 | 2801.2 | -9.77 | periostin, osteoblast specific factor |
| 223614_at | MMP16 | 66.1 | 618.8 | -9.85 | matrix metallopeptidase 16 (membrane-inserted) |
| 225911_at | NPNT | 144.1 | 619.9 | -10.24 | nephronectin |
| 211062_s_at | CPZ | 18.7 | 149.6 | -10.48 | carboxypeptidase Z |
| 203381_s_at | APOE | 121.1 | 1062.8 | -10.56 | apolipoprotein E |
| 202207_at | ARL4C | 97.1 | 1019.6 | -10.80 | ADP-ribosylation factor-like 4C |
| 214677_x_at | IGL@ | 249.4 | 684.2 | -11.06 | immunoglobulin lambda locus |
| 206172_at | IL13RA2 | 59.0 | 191.9 | -11.14 | interleukin 13 receptor, alpha 2 |
| 236859_at | RUNX2 | 11.6 | 205.8 | -11.49 | runt-related transcription factor 2 |
| 215049_x_at | CD163 | 76.8 | 646.4 | -11.67 | CD163 molecule |
| 214438_at | HLX | 5.9 | 149.8 | -12.13 | H2.0-like homeobox |
| 219918_s_at | ASPM | 8.9 | 96.9 | -12.41 | asp (abnormal spindle) homolog, microcephaly associated (Drosophila) |
| 227202_at | CNTN1 | 19.2 | 184.8 | -12.80 | Contactin 1 |
| 230836_at | ST8SIA4 | 72.0 | 375.4 | -13.10 | ST8 alpha-N-acetyl-neuraminide alpha-2,8-sialyltransferase 4 |
| 201292_at | TOP2A | 12.1 | 140.0 | -16.12 | topoisomerase (DNA) II alpha 170kDa |
| 202803_s_at | ITGB2 | 40.0 | 501.0 | -17.02 | integrin, beta 2 (complement component 3 receptor 3 and 4 subunit) |
| 205114_s_at | CCL3 /// CCL3L1 /// CCL3L3 | 24.2 | 400.7 | -17.15 | chemokine (C-C motif) ligand 3 /// chemokine (C-C motif) ligand 3-like 1 /// chemokine (C-C motif) ligand 3-like 3 |
| 206392_s_at | RARRES1 | 24.6 | 423.4 | -19.25 | retinoic acid receptor responder (tazarotene induced) 1 |
| 213603_s_at | RAC2 | 33.3 | 572.2 | -20.00 | ras-related C3 botulinum toxin substrate 2 (rho family, small GTP binding protein Rac2) |
| 203936_s_at | MMP9 | 180.2 | 6715.5 | -21.28 | matrix metallopeptidase 9 (gelatinase B, 92kDa gelatinase, 92kDa type IV collagenase) |
| 213993_at | SPON1 | 22.2 | 288.5 | -22.11 | spondin 1, extracellular matrix protein |
| 221558_s_at | LEF1 | 46.6 | 590.1 | -22.63 | lymphoid enhancer-binding factor 1 |
| 202311_s_at | COL1A1 | 408.5 | 5900.7 | -22.98 | collagen, type I, alpha 1 |
| 209875_s_at | SPP1 | 264.1 | 6536.6 | -26.19 | secreted phosphoprotein 1 |
| 223343_at | MS4A7 | 81.9 | 840.2 | -28.95 | membrane-spanning 4-domains, subfamily A, member 7 |
| 206211_at | SELE | 52.4 | 488.4 | -31.75 | selectin E |
| 207370_at | IBSP | 37.2 | 1883.7 | -34.83 | integrin-binding sialoprotein |
| 209728_at | HLA-DRB4 | 4.0 | 611.2 | -127.02 | major histocompatibility complex, class II, DR beta 4 |
| 205959_at | MMP13 | 11.2 | 3022.7 | -185.25 | matrix metallopeptidase 13 (collagenase 3) |
