# Supplementary material for: Degeneration of Lumbar Intervertebral Discs: Characterization of Anulus Fibrosus Tissue and Cells of Different Degeneration Grades
Source: Int J Mol Sci. 2020 Mar 21;21(6):2165. doi: 10.3390/ijms21062165 (PMC7139657; doi:10.3390/ijms21062165)
Supplement: Supplementary file 1 [file ijms-21-02165-s001.zip › Supplemental Table 2.docx]

Table S2: Differentially regulated genes in monolayer cultivated cells derived from AF tissue of IVDs with mild and severe degeneration grades

| **Affymetrix ID** | **Symbol** | **Mean Signal Mild Degeneration** | **Mean Signal Severe Degeneration** | **Fold Change** | **Name** |
| --- | --- | --- | --- | --- | --- |
| 229151_at | SLC14A1 | 1420.2 | 253.5 | 10.00 | solute carrier family 14 (urea transporter), member 1 (Kidd blood group) |
| 206022_at | NDP | 70.6 | 78.1 | 5.83 | Norrie disease (pseudoglioma) |
| 230493_at | SHISA2 | 318.7 | 429.8 | 4.67 | shisa homolog 2 (Xenopus laevis) |
| 209277_at | TFPI2 | 18.2 | 15.8 | 4.13 | tissue factor pathway inhibitor 2 |
| 202037_s_at | SFRP1 | 181.0 | 247.3 | 4.03 | secreted frizzled-related protein 1 |
| 218755_at | KIF20A | 17.9 | 93.9 | 3.91 | kinesin family member 20A |
| 204641_at | NEK2 | 18.8 | 60.5 | 3.88 | NIMA (never in mitosis gene a)-related kinase 2 |
| 203764_at | DLGAP5 | 14.3 | 58.5 | 3.56 | discs, large (Drosophila) homolog-associated protein 5 |
| 209773_s_at | RRM2 | 31.9 | 159.2 | 3.48 | ribonucleotide reductase M2 |
| 214710_s_at | CCNB1 | 66.4 | 104.6 | 3.27 | cyclin B1 |
| 203554_x_at | PTTG1 | 222.9 | 295.9 | 3.22 | pituitary tumor-transforming 1 |
| 227394_at | NCAM1 | 68.3 | 1034.3 | 3.17 | neural cell adhesion molecule 1 |
| 211003_x_at | TGM2 | 50.6 | 96.0 | 3.13 | transglutaminase 2 (C polypeptide, protein-glutamine-gamma-glutamyltransferase) |
| 226281_at | DNER | 1459.0 | 497.2 | 3.10 | delta/notch-like EGF repeat containing |
| 225544_at | TBX3 | 134.4 | 142.0 | 3.08 | T-box 3 |
| 211343_s_at | COL13A1 | 205.6 | 1454.4 | 2.94 | collagen, type XIII, alpha 1 |
| 219230_at | TMEM100 | 833.7 | 501.3 | 2.94 | transmembrane protein 100 |
| 203231_s_at | ATXN1 | 251.1 | 211.5 | 2.92 | ataxin 1 |
| 218039_at | NUSAP1 | 82.5 | 214.8 | 2.83 | nucleolar and spindle associated protein 1 |
| 222608_s_at | ANLN | 34.5 | 153.6 | 2.76 | anillin, actin binding protein |
| 218542_at | CEP55 | 20.8 | 68.8 | 2.70 | centrosomal protein 55kDa |
| 229121_at | CMKLR1 | 262.1 | 370.5 | 2.68 | chemokine-like receptor 1 |
| 218899_s_at | BAALC | 653.0 | 154.4 | 2.66 | brain and acute leukemia, cytoplasmic |
| 203214_x_at | CDC2 | 24.3 | 70.0 | 2.66 | cell division cycle 2, G1 to S and G2 to M |
| 213599_at | OIP5 | 21.4 | 28.4 | 2.62 | Opa interacting protein 5 |
| 205935_at | FOXF1 | 717.9 | 223.6 | 2.60 | forkhead box F1 |
| 223460_at | CAMKK1 | 270.1 | 129.3 | 2.58 | calcium/calmodulin-dependent protein kinase kinase 1, alpha |
| 225646_at | CTSC | 93.7 | 318.8 | 2.54 | cathepsin C |
| 228033_at | E2F7 | 12.9 | 31.7 | 2.54 | E2F transcription factor 7 |
| 227566_at | NTM | 64.1 | 86.6 | 2.54 | neurotrimin |
| 204026_s_at | ZWINT | 111.9 | 191.0 | 2.48 | ZW10 interactor |
| 222848_at | CENPK | 50.8 | 122.5 | 2.44 | centromere protein K |
| 219134_at | ELTD1 | 348.4 | 960.5 | 2.41 | EGF, latrophilin and seven transmembrane domain containing 1 |
| 213338_at | TMEM158 | 63.5 | 152.5 | 2.41 | transmembrane protein 158 |
| 206631_at | PTGER2 | 424.3 | 158.6 | 2.39 | prostaglandin E receptor 2 (subtype EP2), 53kDa |
| 210997_at | HGF | 15.3 | 25.6 | 2.32 | hepatocyte growth factor (hepapoietin A; scatter factor) |
| 213661_at | PAMR1 | 250.0 | 280.0 | 2.24 | peptidase domain containing associated with muscle regeneration 1 |
| 204115_at | GNG11 | 1945.4 | 2188.7 | 2.23 | guanine nucleotide binding protein (G protein), gamma 11 |
| 1554768_a_at | MAD2L1 | 27.6 | 63.1 | 2.23 | MAD2 mitotic arrest deficient-like 1 (yeast) |
| 217979_at | TSPAN13 | 1766.7 | 1349.2 | 2.23 | tetraspanin 13 |
| 204033_at | TRIP13 | 37.3 | 77.1 | 2.21 | thyroid hormone receptor interactor 13 |
| 226210_s_at | MEG3 | 560.5 | 161.9 | 2.19 | maternally expressed 3 (non-protein coding) |
| 227314_at | ITGA2 | 133.3 | 450.1 | 2.18 | integrin, alpha 2 (CD49B, alpha 2 subunit of VLA-2 receptor) |
| 223339_at | ATPIF1 | 226.3 | 123.5 | 2.16 | ATPase inhibitory factor 1 |
| 201920_at | SLC20A1 | 2118.7 | 1520.7 | 2.16 | solute carrier family 20 (phosphate transporter), member 1 |
| 228559_at | CENPN | 57.9 | 64.6 | 2.14 | centromere protein N |
| 204170_s_at | CKS2 | 132.0 | 362.9 | 2.14 | CDC28 protein kinase regulatory subunit 2 |
| 238762_at | MTHFD2L | 91.4 | 78.2 | 2.14 | methylenetetrahydrofolate dehydrogenase (NADP+ dependent) 2-like |
| 213865_at | DCBLD2 | 26.4 | 5.1 | 2.08 | discoidin, CUB and LCCL domain containing 2 |
| 219588_s_at | NCAPG2 | 72.3 | 94.8 | 2.06 | non-SMC condensin II complex, subunit G2 |
| 223093_at | ANKH | 1373.2 | 793.9 | 2.03 | ankylosis, progressive homolog (mouse) |
| 213226_at | CCNA2 | 85.3 | 84.5 | 2.02 | cyclin A2 |
| 209897_s_at | SLIT2 | 104.6 | 424.4 | 2.02 | slit homolog 2 (Drosophila) |
| 48808_at | DHFR | 124.2 | 267.7 | 2.00 | dihydrofolate reductase |
| 203349_s_at | ETV5 | 292.3 | 315.6 | 2.00 | ets variant 5 |
| 209228_x_at | TUSC3 | 136.0 | 299.6 | 2.00 | tumor suppressor candidate 3 |
| 202986_at | ARNT2 | 32.9 | 39.2 | -2.00 | aryl-hydrocarbon receptor nuclear translocator 2 |
| 204040_at | RNF144A | 211.1 | 525.3 | -2.00 | ring finger protein 144A |
| 209822_s_at | VLDLR | 143.1 | 126.4 | -2.00 | very low density lipoprotein receptor |
| 209185_s_at | IRS2 | 1289.5 | 912.4 | -2.03 | insulin receptor substrate 2 |
| 207172_s_at | CDH11 | 118.3 | 810.4 | -2.05 | cadherin 11, type 2, OB-cadherin (osteoblast) |
| 225922_at | FNIP2 | 1844.3 | 1934.9 | -2.05 | folliculin interacting protein 2 |
| 228469_at | PPID | 91.6 | 98.7 | -2.05 | Peptidylprolyl isomerase D |
| 232865_at | AFF4 | 240.4 | 363.2 | -2.06 | AF4/FMR2 family, member 4 |
| 208733_at | RAB2A | 86.9 | 89.0 | -2.06 | RAB2A, member RAS oncogene family |
| 232183_at | SERAC1 | 88.4 | 121.0 | -2.06 | serine active site containing 1 |
| 204881_s_at | UGCG | 309.0 | 432.9 | -2.06 | UDP-glucose ceramide glucosyltransferase |
| 204984_at | GPC4 | 79.5 | 433.9 | -2.08 | glypican 4 |
| 203088_at | FBLN5 | 114.0 | 229.5 | -2.09 | fibulin 5 |
| 203725_at | GADD45A | 859.0 | 1380.2 | -2.09 | growth arrest and DNA-damage-inducible, alpha |
| 203886_s_at | FBLN2 | 117.8 | 190.7 | -2.13 | fibulin 2 |
| 221577_x_at | GDF15 /// LOC100292463 | 11.1 | 64.7 | -2.13 | growth differentiation factor 15 /// similar to growth differentiation factor 15 |
| 203865_s_at | ADARB1 | 824.2 | 659.4 | -2.16 | adenosine deaminase, RNA-specific, B1 (RED1 homolog rat) |
| 228790_at | FAM110B | 146.6 | 230.0 | -2.16 | family with sequence similarity 110, member B |
| 202971_s_at | DYRK2 | 127.0 | 179.6 | -2.18 | dual-specificity tyrosine-(Y)-phosphorylation regulated kinase 2 |
| 230083_at | USP53 | 1201.5 | 333.2 | -2.18 | ubiquitin specific peptidase 53 |
| 202995_s_at | FBLN1 | 700.7 | 1131.6 | -2.19 | fibulin 1 |
| 228399_at | OSR1 | 6.3 | 9.4 | -2.19 | odd-skipped related 1 (Drosophila) |
| 225987_at | STEAP4 | 447.2 | 1962.3 | -2.19 | STEAP family member 4 |
| 218532_s_at | FAM134B | 2162.8 | 988.0 | -2.21 | family with sequence similarity 134, member B |
| 209355_s_at | PPAP2B | 171.7 | 260.7 | -2.23 | phosphatidic acid phosphatase type 2B |
| 204456_s_at | GAS1 | 125.1 | 95.4 | -2.24 | growth arrest-specific 1 |
| 235085_at | PRAGMIN | 42.8 | 195.7 | -2.24 | homolog of rat pragma of Rnd2 |
| 227529_s_at | AKAP12 | 41.4 | 70.6 | -2.26 | A kinase (PRKA) anchor protein 12 |
| 213348_at | CDKN1C | 978.8 | 905.2 | -2.26 | cyclin-dependent kinase inhibitor 1C (p57, Kip2) |
| 222908_at | FAM38B | 196.0 | 302.8 | -2.28 | family with sequence similarity 38, member B |
| 227911_at | ARHGAP28 | 56.4 | 168.5 | -2.32 | Rho GTPase activating protein 28 |
| 225283_at | ARRDC4 | 379.3 | 540.4 | -2.32 | arrestin domain containing 4 |
| 225436_at | FAM108C1 | 53.2 | 138.0 | -2.33 | family with sequence similarity 108, member C1 |
| 229518_at | FAM46B | 172.5 | 108.7 | -2.35 | family with sequence similarity 46, member B |
| 213125_at | OLFML2B | 386.3 | 2864.7 | -2.35 | olfactomedin-like 2B |
| 209193_at | PIM1 | 355.9 | 343.0 | -2.35 | pim-1 oncogene |
| 201008_s_at | TXNIP | 800.6 | 790.7 | -2.39 | thioredoxin interacting protein |
| 212651_at | RHOBTB1 | 334.4 | 469.7 | -2.41 | Rho-related BTB domain containing 1 |
| 219949_at | LRRC2 | 279.5 | 116.4 | -2.42 | leucine rich repeat containing 2 |
| 206025_s_at | TNFAIP6 | 2033.5 | 1603.7 | -2.42 | tumor necrosis factor, alpha-induced protein 6 |
| 209348_s_at | MAF | 784.9 | 1214.2 | -2.46 | v-maf musculoaponeurotic fibrosarcoma oncogene homolog (avian) |
| 212805_at | PRUNE2 | 930.7 | 647.4 | -2.46 | prune homolog 2 (Drosophila) |
| 217678_at | SLC7A11 | 15.9 | 21.8 | -2.46 | solute carrier family 7, (cationic amino acid transporter, y+ system) member 11 |
| 213006_at | CEBPD | 228.6 | 179.5 | -2.48 | CCAAT/enhancer binding protein (C/EBP), delta |
| 202157_s_at | CUGBP2 | 257.4 | 561.4 | -2.48 | CUG triplet repeat, RNA binding protein 2 |
| 218574_s_at | LMCD1 | 319.9 | 383.9 | -2.48 | LIM and cysteine-rich domains 1 |
| 207692_s_at | ACAN | 4940.2 | 1346.5 | -2.52 | aggrecan |
| 210764_s_at | CYR61 | 4663.4 | 2568.7 | -2.52 | cysteine-rich, angiogenic inducer, 61 |
| 202481_at | DHRS3 | 697.7 | 266.6 | -2.52 | dehydrogenase/reductase (SDR family) member 3 |
| 203083_at | THBS2 | 3075.1 | 4736.0 | -2.52 | thrombospondin 2 |
| 202709_at | FMOD | 24907.0 | 7901.3 | -2.54 | fibromodulin |
| 202761_s_at | SYNE2 | 584.4 | 783.0 | -2.54 | spectrin repeat containing, nuclear envelope 2 |
| 209396_s_at | CHI3L1 | 496.3 | 1775.4 | -2.58 | chitinase 3-like 1 (cartilage glycoprotein-39) |
| 232568_at | MGC24103 | 338.7 | 389.4 | -2.60 | hypothetical MGC24103 |
| 212977_at | CXCR7 | 7473.3 | 3769.7 | -2.64 | chemokine (C-X-C motif) receptor 7 |
| 219371_s_at | KLF2 | 3796.3 | 2340.3 | -2.64 | Kruppel-like factor 2 (lung) |
| 223218_s_at | NFKBIZ | 4618.7 | 3916.9 | -2.66 | nuclear factor of kappa light polypeptide gene enhancer in B-cells inhibitor, zeta |
| 220327_at | VGLL3 | 72.5 | 93.7 | -2.66 | vestigial like 3 (Drosophila) |
| 1555827_at | CCNL1 | 195.4 | 262.7 | -2.79 | Cyclin L1 |
| 238049_at | GRAMD3 | 215.7 | 102.2 | -2.79 | GRAM domain containing 3 |
| 233261_at | EBF1 | 98.2 | 127.8 | -2.83 | Early B-cell factor 1 |
| 228707_at | CLDN23 | 58.8 | 55.9 | -2.85 | claudin 23 |
| 226189_at | ITGB8 | 330.8 | 487.4 | -2.89 | integrin, beta 8 |
| 235879_at | MBNL1 | 1366.5 | 676.9 | -2.89 | Muscleblind-like (Drosophila) |
| 210135_s_at | SHOX2 | 214.7 | 316.5 | -2.94 | short stature homeobox 2 |
| 204430_s_at | SLC2A5 | 167.8 | 301.3 | -2.94 | solute carrier family 2 (facilitated glucose/fructose transporter), member 5 |
| 201842_s_at | EFEMP1 | 1411.0 | 1195.6 | -3.05 | EGF-containing fibulin-like extracellular matrix protein 1 |
| 203139_at | DAPK1 | 215.8 | 299.6 | -3.15 | death-associated protein kinase 1 |
| 235564_at | ZNF117 | 26.3 | 70.3 | -3.15 | Zinc finger protein 117 |
| 203434_s_at | MME | 32.5 | 109.9 | -3.25 | membrane metallo-endopeptidase |
| 214091_s_at | GPX3 | 11915.1 | 1856.7 | -3.27 | glutathione peroxidase 3 (plasma) |
| 210319_x_at | MSX2 | 93.6 | 80.6 | -3.27 | msh homeobox 2 |
| 217525_at | OLFML1 | 142.1 | 317.7 | -3.27 | olfactomedin-like 1 |
| 206101_at | ECM2 | 4303.3 | 2966.5 | -3.32 | extracellular matrix protein 2, female organ and adipocyte specific |
| 209167_at | GPM6B | 295.8 | 725.0 | -3.32 | glycoprotein M6B |
| 226736_at | CHURC1 | 662.0 | 807.7 | -3.38 | churchill domain containing 1 |
| 219304_s_at | PDGFD | 847.8 | 1124.9 | -3.40 | platelet derived growth factor D |
| 213247_at | SVEP1 | 107.7 | 511.5 | -3.46 | sushi, von Willebrand factor type A, EGF and pentraxin domain containing 1 |
| 40665_at | FMO3 | 38.5 | 173.1 | -3.59 | flavin containing monooxygenase 3 |
| 205666_at | FMO1 | 46.3 | 164.7 | -3.62 | flavin containing monooxygenase 1 |
| 221111_at | IL26 | 3.3 | 6.7 | -3.70 | interleukin 26 |
| 217767_at | C3 | 51.8 | 160.4 | -3.76 | complement component 3 |
| 204774_at | EVI2A | 1513.2 | 1464.6 | -3.76 | ecotropic viral integration site 2A |
| 218918_at | MAN1C1 | 135.3 | 226.0 | -3.76 | mannosidase, alpha, class 1C, member 1 |
| 227062_at | NEAT1 | 500.3 | 414.9 | -3.79 | nuclear paraspeckle assembly transcript 1 (non-protein coding) |
| 212841_s_at | PPFIBP2 | 263.9 | 369.4 | -3.97 | PTPRF interacting protein, binding protein 2 (liprin beta 2) |
| 203666_at | CXCL12 | 461.3 | 2751.7 | -4.00 | chemokine (C-X-C motif) ligand 12 (stromal cell-derived factor 1) |
| 229759_s_at | VEPH1 | 5.1 | 8.2 | -4.06 | ventricular zone expressed PH domain homolog 1 (zebrafish) |
| 202149_at | NEDD9 | 683.1 | 849.6 | -4.29 | neural precursor cell expressed, developmentally down-regulated 9 |
| 203475_at | CYP19A1 | 20.6 | 26.0 | -4.32 | cytochrome P450, family 19, subfamily A, polypeptide 1 |
| 205027_s_at | MAP3K8 | 538.8 | 420.8 | -4.32 | mitogen-activated protein kinase kinase kinase 8 |
| 203889_at | SCG5 | 32.8 | 34.8 | -4.32 | secretogranin V (7B2 protein) |
| 220313_at | GPR88 | 28.1 | 18.5 | -4.35 | G protein-coupled receptor 88 |
| 206618_at | IL18R1 | 10.2 | 43.9 | -4.85 | interleukin 18 receptor 1 |
| 220351_at | CCRL1 | 75.6 | 57.7 | -4.92 | chemokine (C-C motif) receptor-like 1 |
| 238584_at | IQCA1 | 198.1 | 70.7 | -5.12 | IQ motif containing with AAA domain 1 |
| 229271_x_at | COL11A1 | 1307.6 | 1137.1 | -5.16 | collagen, type XI, alpha 1 |
| 223044_at | SLC40A1 | 2556.6 | 3496.3 | -5.16 | solute carrier family 40 (iron-regulated transporter), member 1 |
| 200986_at | SERPING1 | 3816.5 | 2320.5 | -5.32 | serpin peptidase inhibitor, clade G (C1 inhibitor), member 1 |
| 213832_at | KCND3 | 81.2 | 83.5 | -5.53 | potassium voltage-gated channel, Shal-related subfamily, member 3 |
| 213764_s_at | MFAP5 | 121.9 | 98.9 | -5.61 | microfibrillar associated protein 5 |
| 224724_at | SULF2 | 1791.3 | 1496.8 | -5.61 | sulfatase 2 |
| 204052_s_at | SFRP4 | 169.7 | 767.7 | -5.70 | secreted frizzled-related protein 4 |
| 219505_at | CECR1 | 123.4 | 300.5 | -6.35 | cat eye syndrome chromosome region, candidate 1 |
| 238720_at | OMG | 34.8 | 42.1 | -8.19 | Oligodendrocyte myelin glycoprotein |
| 219087_at | ASPN | 20240.5 | 8186.0 | -9.40 | asporin |
| 228268_at | FMO2 | 80.7 | 144.2 | -9.48 | flavin containing monooxygenase 2 (non-functional) |
| 1558668_s_at | SPATA22 | 15.2 | 24.0 | -10.48 | spermatogenesis associated 22 |
